# A Systematic Review of the Relationships between Intimate Partner Violence and HIV/AIDS

**DOI:** 10.1371/journal.pone.0081044

**Published:** 2013-11-25

**Authors:** Fiona G. Kouyoumdjian, Nicole Findlay, Michael Schwandt, Liviana M. Calzavara

**Affiliations:** Dalla Lana School of Public Health, University of Toronto, Toronto, Ontario, Canada; Rollins School of Public Health, United States of America

## Abstract

**Background:**

Intimate partner violence (IPV) is a significant health problem that has been associated with HIV infection in numerous studies. We aimed to systematically review the literature on relationships between IPV and HIV in order to describe the prevalence of IPV in people with HIV, the prevalence of HIV in people experiencing IPV, the association between IPV and HIV, and evidence regarding mechanisms of risk and interventions.

**Methods:**

Data sources were 10 electronic databases and reference lists. Studies were included if they reported data on the relationship between IPV and HIV. All records were independently reviewed by two authors at the stages of title and abstract review and full text review. Any abstract considered eligible by either reviewer was reviewed in full, and any disagreement regarding eligibility of full texts or data extracted was resolved by discussion.

**Results:**

101 articles were included. Experiencing IPV and HIV infection were associated in unadjusted analyses in most studies, as well as in adjusted analyses in many studies. The findings of qualitative and quantitative studies assessing potential mechanisms linking IPV and HIV were variable. Few interventions have been assessed, but two identified in this review were promising in terms of preventing IPV, though not HIV infection.

**Conclusions:**

Experiencing IPV and HIV infection tend to be associated in unadjusted analyses, suggesting that IPV screening and linkage with relevant programs and services may be valuable. It is unclear whether there is a causal association between experiencing IPV and HIV infection. Research should focus on defining parameters of IPV which are relevant to HIV infection, including type of IPV and period of exposure and risk, on assessing potential mechanisms, and on developing and assessing interventions which build on the strengths of existing studies.

## Introduction

Intimate partner violence (IPV) is defined as “behaviour within an intimate relationship that causes physical, sexual, or psychological harm, including acts of physical aggression, sexual coercion, psychological abuse and controlling behaviours” [1]. IPV has been associated with significant morbidity and mortality worldwide, and is a serious public health and human rights problem [2]. In the past two decades, theoretical and empirical evidence has emerged regarding an association between IPV and HIV infection [3,4], with relevance for public health and clinical practice, policy development, and research. Clearly elucidating this relationship is important to ensure that efforts are optimized in the prevention of the IPV and HIV epidemics.

While other reviews have been conducted of the association between IPV and HIV [3-10], they have been limited by a specific geographic focus [5,6,10], not including gray literature [3-10] or qualitative studies [3-10], and not including studies conducted in the last several years. These limitations preclude a comprehensive description of the state of knowledge about this relationship. 

We systematically reviewed the literature on relationships between IPV and HIV to summarize the prevalence of IPV in people with HIV, the prevalence of HIV in people experiencing IPV, and data on the association between IPV and HIV, and to describe evidence regarding mechanisms of risk and interventions.

## Methods

For this study, we used the definition of IPV from the World Health Organization (provided above) [1], and we included violence by a current or former partner or spouse, whether cohabitating or not. Inclusion criteria were original research reporting: 1) data on the relationship between IPV and HIV; 2) data about the mechanisms of association between IPV and HIV; or, 3) theoretical discussion of the relationships between IPV and HIV. Studies had to specify their methods in order to be considered original research; reviews and commentaries were excluded. Exclusion criteria were studies which 1) looked at the association between IPV and HIV risk factors (instead of HIV infection); or 2) assessed violence only in commercial sex work or in non-intimate relationships, e.g. sexual assault by a stranger. 

Databases searched were Medline, PsycINFO, CINAHL, Cochrane Database of Systematic Reviews, Sociological Abstracts, Embase, Web of Science, PapersFirst, ProceedingsFirst, and ProQuest Dissertations and Theses, using a search strategy of text words and indexing terms. No language or date limits were applied. The search was conducted in September 2011 for all databases except Sociological Abstracts, for which the search was conducted in December 2011. Reference lists of review articles identified in the search and eligible articles were also hand searched. In addition, authors identified other eligible articles. 

In an initial review, two authors independently reviewed all titles and abstracts identified in the search. Full texts were retrieved if either reviewer determined that they were eligible for review. Two authors then independently reviewed each full text to assess eligibility, and for those deemed eligible, to extract relevant data. Disagreements regarding eligibility and extracted data were resolved through discussion. A hierarchy was used to classify the reason for excluding texts: 1) if no original data were included, this was classified as the reason, 2) if original data were included but no data on IPV were included, not about IPV was classified as the reason, 3) if original data on IPV were included but no data on HIV were included, not about HIV was classified as the reason, and 4) if original data on HIV and on IPV were included, but there were no data on the association between IPV and HIV, this was classified as the reason. When more than one record or paper was identified with data from the same study, all papers were included if they presented different data, or the paper with the earlier date was included if they presented the same data.

Data extraction forms were piloted by two authors and modified accordingly. Data extracted included study characteristics: study publication year, study period in years, study location, i.e. city, state, and country, study site, e.g. clinic, community-based, school-based, or other, and study type, i.e. cohort, cross-sectional study, *etc*.; data regarding IPV: definition, period of exposure, whether experienced or perpetrated; potential sources of bias; and qualitative or quantitative data regarding association. All direct quotations from participants in qualitative studies were included if they described the association between IPV and HIV. For assessment of bias in quantitative studies, components of a public health critical appraisal tool [11] were considered, with particular focus on selection of participants, measurement of exposure, and handling of confounders. Type of IPV was categorized as physical, sexual, or verbal, with verbal including verbal, psychological, or emotional IPV, or control. 

## Results

**Figure 1:**
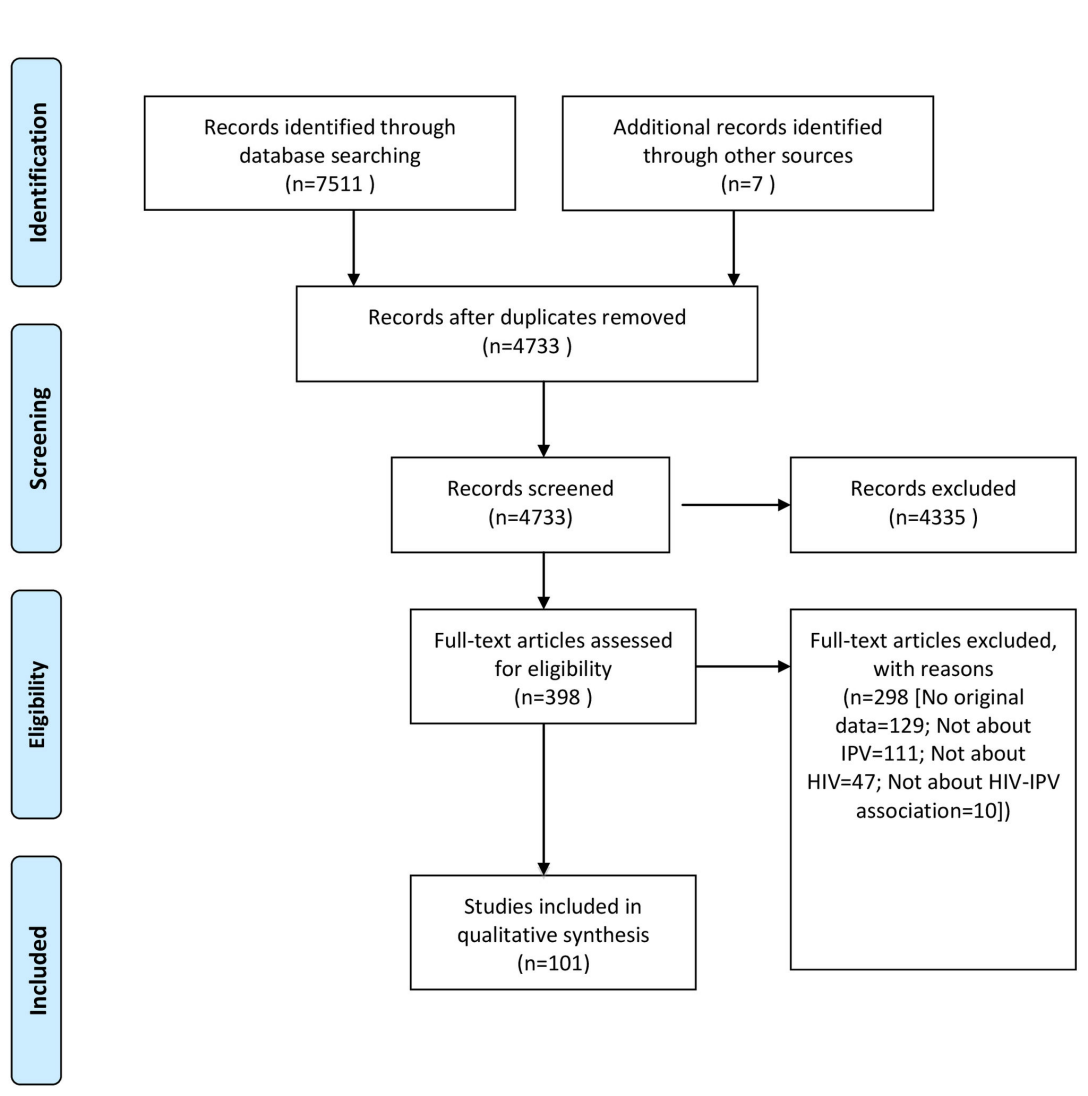
Flow diagram.

As shown in Figure 1, our search identified 4733 unique records. We assessed 398 full-text articles for eligibility, and included 101 in the qualitative synthesis [12-111]. Included studies are shown in Table 1. Of the 101 studies, only 31 included data on all three categories of IPV: physical IPV, sexual IPV, and verbal IPV. Forty-five studies were conducted in North America, 44 in Africa, nine in Asia, two in South America, and one in several developing countries in more than one region. Most studies focused on the perspective of people who had experienced IPV. 

**Table 1:** Studies included in qualitative synthesis.

**Region**	**Article**	**Data^*a*^**	**Type of IPV^*b*^**			**Perspective^*c*^**	**Region**	**Article**	**Data^*a*^**	**Type of IPV^*b*^**			**Perspective^*c*^**
			**P**	**S**	**V**					**P**	**S**	**V**	
Africa	Amuyunzu-Nyamongo et al., 2007	Mx		✓		E	S America	Barros et al., 2011	Ql	✓	✓	✓	E
	Apondi et al., 2007	Qn	✓			E		Porto et al., 2003	Ql	✓	✓		E
	Dude, 2011	Qn	✓	✓	✓	E	Multi**^*d*^**	Harling et al., 2010	Qn	✓	✓		E
	Dunkle et al., 2004	Qn	✓	✓		E	N America	Bartholomew et al., 2008	Qn	✓		✓	B
	Eloff et al., 2011	Mx	✓		✓	E		Bogart et al., 2005	Qn	✓	✓	✓	B
	Emusu et al., 2009	Qn	✓	✓	✓	E		Brady et al., 2002	Qn		✓		E
	Ezeanochie et al., 2011	Qn	✓	✓	✓	E		Burke et al., 2005	Qn	✓	✓		E
	Ezechi et al., 2009	Qn	✓	✓	✓	E		Campbell, 2009	Qn			✓	E
	Fonck et al., 2005	Qn	✓	✓		E		Craft et al., 2005	Qn	✓	✓	✓	B
	Fox et al., 2007	Ql	✓	✓	✓	E		Davila et al., 2008	Qn	-	-	-	E
	Francisco, 2010	Mx	✓	✓	✓	B		DiStefano et al., 2011	Ql	-	-	-	B
	Gaillard et al., 2002	Qn	-	-	-	E		Frye et al., 2007	Qn	✓	✓		P
	Jewkes et al., 2006	Qn	✓	✓		E		Galvan et al., 2004	Qn	✓	✓		B
	Jewkes et al., 2008	Qn	-	-	-	P		Gard et al., 2002	Qn	✓			E
	Jewkes et al., 2010	Qn	✓	✓		E		Gielen et al., 1997	Ql	✓		✓	E
	Jewkes et al., 2011	Qn	✓	✓		P		Gielen et al., 2000	Mx	✓	✓	✓	E
	Karamagi et al, 2006	Mx	✓	✓		E		Gielen et al., 2002	Qn	✓	✓	✓	E
	Ketchen et al., 2009	Qn			✓	E		Gonzalez-Guarda et al., 2010	Ql	✓	✓	✓	B
	Kiarie et al., 2006	Qn	✓		✓	B		Greenwood et al., 2002	Qn	✓	✓	✓	E
	Kouyoumdjian et al., 2013	Qn	✓	✓	✓	E		Hamburger et al., 2004	Qn	✓		✓	E
	Makayoto et al., 2012	Qn	✓	✓	✓	E		Henny et al., 2007	Qn	✓			E
	Maman, 2000	Qn	✓	✓	✓	B		Herman et al., 2004	Ql	✓		✓	E
	Maman et al., 2002	Qn	✓	✓	✓	E		Illangasekare et al., 2010	Qn	✓		✓	E
	Mattson et al., 2009	Qn		✓		E		Jones et al., 2003	Qn	✓		✓	E
	Mayanja, 2010	Qn	✓	✓	✓	E		Kalokhe et al., 2011	Qn	-	-	-	E
	Mukanyonga et al., 2011	Qn		✓		E		Koenig et al., 2002	Qn	✓		✓	E
	Muldoon et al., 2011	Ql	-	-	-	E		Lang et al., 2007	Qn	✓	✓		E
	Murray et al., 2006	Ql	✓			E		Laughon et al., 2007	Qn	✓	✓		E
	Ntaganira et al., 2008	Qn	✓		✓	E		Lichtenstein, 2004	Ql	✓	✓	✓	E
	Ntaganira et al., 2009	Qn	✓		✓	E		McDonnell et al., 2003	Mx	✓	✓	✓	E
	Pettifor et al., 2004	Qn		✓	✓	E		Moreno, 2007	Ql	-	-	-	E
	Phorano et al., 2005	Ql	✓	✓	✓	E		Nava, 2010	Qn	✓	✓		E
	Prabhu et al., 2011	Qn	✓	✓		E		Neundorfer et al., 2005	Ql	-	-	-	E
	Pronyk et al., 2006	Qn	✓	✓		E		Newcomb et al., 2004	Qn	✓			E
	Sa et al., 2008	Qn	✓	✓		E		O'Campo et al., 2002	Qn	✓	✓		E
	Semrau et al., 2005	Qn	✓		✓	E		Pantalone, 2007	Mx	✓	✓	✓	E
	Shamu et al., 2011	Ql		✓		B		Ramachandran et al., 2010	Qn	✓	✓	✓	E
	Shi et al., 2013	Qn	✓	✓	✓	E		Reading et al., 2009	Ql		✓	✓	E
	Strebel et al., 2006	Ql	✓	✓	✓	B		Sareen et al., 2009	Qn	✓	✓		E
	van der Straten et al., 1995	Qn	✓	✓		B		Shelton et al., 2005	Qn	✓	✓		E
	van der Straten et al., 1998	Qn	✓	✓		E		Sherbourne et al., 2003	Qn	✓			E
	Were et al., 2011	Qn	✓	✓	✓	E		Siemieniuk et al., 2010	Qn	✓	✓	✓	E
	Winchester, 2011	Mx	✓	✓	✓	E		Sormanti et al., 2008	Qn	✓	✓		E
	Zablotska et al., 2009	Qn	✓	✓	✓	E		Stevens et al., 2007	Ql	✓		✓	E
Asia	Chandrasekaran et al., 2007	Qn	✓	✓	✓	E		Ulibarri et al., 2010	Qn	✓	✓	✓	E
	Dayaprasad et al., 2010	Qn	✓	✓		E		Wiliams et al., 2008	Qn	✓			E
	Decker et al., 2009	Qn	✓	✓		B		Wright et al., 2006	Qn	-	-	-	E
	Ghosh et al., 2011	Qn		✓		E		Zabler, 2009	Ql	✓	✓	✓	B
	Go et al., 2003	Ql	✓	✓	✓	E	Asia	Lewis et al., 2008	Mx	✓	✓	✓	E
	Gupta et al., 2008	Qn	✓		✓	E		Silverman et al., 2008	Qn	✓	✓		E
	Kulkarni et al., 2009	Qn	✓	✓		E							

***^a^***Type of study: mIxed methods (Mx); qualitative (Ql); quantitative (Qn). **^*b*^**Indicates whether the study specifically addressed physical IPV (P), sexual IPV (S) and/or verbal, emotional or psychological IPV or control (V). Studies that did not specify the type of IPV were noted as "-". **^*c*^**Perspective refers to whether the study investigated IPV that was experienced (E), perpetrated (P) or both (B). **^*d*^**This study was conducted in several developing countries encompassing more than one world region.

### 1: Prevalence of experiencing and perpetrating IPV in people with HIV

Studies conducted in diverse settings reveal that the prevalence of experiencing IPV is often high in people with HIV, and also that many people with HIV have experienced several types of IPV. These data are presented in Table 2, which shows the prevalence of having experienced IPV in studies of people with HIV [16,17,21,29,35,45,59,67,69,70,77,86,88,96,97], and Table 3, which shows the prevalence of having experienced IPV by HIV status [14,37,41-43,55,56,60,68,72,76,82,94]. 

**Table 2:** Prevalence of having experienced intimate partner violence in studies of people with HIV.

Region	Article	Population**^*a*^**	N	Age**^*b*^**	IPV prevalence**^*c*^**
North America	Bogart et al., 2005**^*d*^**	WSM	286	36.3 (0.66)	P/S/V: 19.8%
		Heterosexual men	148	43.3 (0.69)	P/S/V: 24.2%
		MSM	292	38.8 (0.39)	P/S/V: 16.7%
	Brady et al., 2002	WSM	100	38**^*e*^**	S: 40%
	Craft et al., 2005	MSM	51	25-63	P^f^: 39.2%
					P^g^: 23.5%
					S: 33.3%
					V: 72.5%
	Galvan et al., 2004	MSM, Heterosexual men & WSM	724	≥ 18	P/S: 19.7%
	Henny et al., 2007	Homeless**^*h*^** & M/F/T	644	16-63	P: 46.4%
		Homeless**^*h*^** & M		16-63	P: 38.8%
		Homeless**^*h*^** & F		16-63	P: 62.2%
		Homeless**^*h*^** & T		16-63	P: 66.7%
	Lang et al., 2007	African American**^*i*^** & WSM	304	18-50	P/S: 10.2%
				18-50	P: 9.9%
				18-50	S: 1.3%
	Nava et al., 2010	WSM	272	23-72	P/S: 52%
	Sherbourne et al., 2003**^*d*^**	WSM	847	36.3**^*e*^**	P: 20%
	Siemieniuk et al., 2010	Overall study population	853	-	P/S/V: 14%
		Heterosexual women	186	-	P/S/V: 34%
		Gay men	399	-	P/S/V: 22%
		Bisexual men & women	57	-	P/S/V: 30%
		Heterosexual men	205	-	P/S/V: 14%
		Aboriginal	59	-	P/S/V: 46%
		Black	167	-	P/S/V: 16%
		Caucasian	208	-	P/S/V: 24%
	Wright et al., 2006	WSM & MSW	102	-	36.4%**^*j*^**
		MSM		-	11.1%**^*j*^**
South America	Porto et al., 2003	Black/Brown Brazilian WSM	57	17-42	P/S/V: 28%
Sub-Saharan Africa	Ezeanochie et al., 2011	Pregnant WSM	305	30.9 (4.2)	P/S/V: 32.5%
					P: 5.9%
					S: 9.8%
					V: 27.5%
	Mayanja et al., 2010	WSM	178	-	S+V: 31.5%
					S: 36%
					V: 60%
		MSW	117	-	S: <3%
					V: 49%
	Mukanyonga et al., 2011	WSM	382	-	S: 25.9%
					P: 39.3%
	Winchester et al, 2011	WSM	200	18-65	S: 42.3%

***^a^***Populations to which the estimates apply: WSM = women who have sex with men, MSW = men who have sex with women, MSM = men who have sex with men, M = male, F = female, T = transgender. **^*b*^**Age range or mean age (standard deviation). **^*c*^**P = physical IPV, S = sexual IPV, V = verbal, psychological or emotional IPV or control. **^*d*^**These two articles both present data from the HIV Cost and Services Utilization Study. **^*e*^**Mean age only, standard deviation not reported. **^*f*^**Physical IPV defined as being physical assaulted by a partner. **^*g*^**Physical IPV defined as physical injuries suffered from physical assault by a partner that required medical attention and involved persistent pain and/or resulted in bone and tissue damage. **^*h*^**This population was either homeless or at severe risk of homelessness. **^*i*^**Population >75% African American **^*j*^**The types of IPV represented in this article is not clear.

**Table 3:** Prevalence of having experienced intimate partner violence by HIV status.

Region	Article	Population**^*a*^**	N	Age**^*b*^**	IPV prevalence (95% CI)		
					HIV+**^*c*^**	HIV-**^*c*^**	p value**^*d*^**
Asia	Ghosh et al., 2011	WSM & MSW	37781	15-54	S: 9%	-	-
	Gupta et al., 2008	WSM	459	31.6 (7.3)	P: 0.20%**^*e*^**	P: 0.11%**^*e*^**	<0.01
					V: 43.5%**^*f*^**	V: 40.7%**^*f*^**	NS
					V: 39.3%**^*g*^**	V: 30.2%**^*g*^**	<0.05
North America	Greenwood et al., 2002	MSM	2881	≥18	P/S/V: 43% (38-49)	P/S/V: 40% (38-43)	-
					P: 29% (24 - 34)	P: 21% (19 - 24)	-
					S: 6.4% (3.6 - 11.1)	S: 4.9% (3.7-6.4)	-
					V: 39% (33-44)	V: 35% (32-37)	-
	Hamburger et al., 2004	WSM	403	34**^*h*^**	P: 28%	P: 14%	-
					V: 67%	V: 65%	-
	Koenig et al., 2002	Pregnant WSM	634	27.8**^*h*^**	P: 5.1%	P: 5.8%	0.72
					V: 14.5%	V: 17.4%	0.36
	Laughon et al., 2007	African American & WSM	445	≥18	P/S: 38%	P/S: 33%	NS
	McDonnell et al., 2003	African American & WSM	611	≥18	P/S: 41%	P/S: 47%	-
					P+S: 18%	P+S: 19%	-
					P: 56%	P: 64%	-
					S: 20%	S: 22%	-
					V: 55%	V: 53%	-
South America	Barros et al., 2011	Non-Black/Brown Brazilian & WSM	3193	15-49	P/S/V: 72.1%	P/S/V: 43.5%	-
					V: 11.2%	V: 15.7%	-
Sub-Saharan Africa	Kiarie et al., 2006	Pregnant WSM & MSW	2836	-	P/V: 37%	P/V: 26%	<0.001
	Ntanganira et al., 2008	Pregnant WSM	600	18-47	P: 44.3%	P: 20.3%	-
	Pettifor et al., 2004	WSM	4066	15-24	S: 3.6%	S: 3.9%	0.82
	Sa et al., 2008	WSM	1418	20-44	P/S: 25.2%	-	-
	Were et al., 2011	WSM	3408**^*i*^**	≥18	P/S/V: 2.8%	P/S/V: 3.1%	-
		MSW	3408**^*i*^**	≥18	P/S/V: 2.0%	P/S/V: 1.0%	-

***^a^***Populations to which the estimates apply: WSM = women who have sex with men, MSW = men who have sex with women. **^*b*^**Age range or mean age (standard deviation). **^*c*^**P = physical IPV, S = sexual IPV, V = verbal, psychological or emotional IPV or control. **^*d*^**NS = not significant. **^*e*^**This estimate defines physical IPV as answering yes to “ever hit, kicked or punched by husband”. **^*f*^**This estimate defines verbal, psychological or emotional IPV or control as answering yes to “relationship quality coercive”. **^*g*^**This estimate defines verbal, psychological or emotional IPV or control as answering yes to “ever forced/coerced in marriage”. **^*h*^**Mean age only, standard deviation not reported. **^*i*^**This N refers to 3408 serodiscordant couples, in 2299 of which the HIV-infected partner was female.

Three studies also reported the frequency of IPV perpetration in people with HIV. In the HIV Cost and Services Utilization Study [35], a national study in the USA of HIV-positive persons in 1998, 20% of participants had perpetrated physical or sexual IPV in the past six months, and in 48% of abusive intimate relationships, abuse was mutual, i.e. was perpetrated by both partners. Another analysis of data from the same study [16] revealed that 24.9% of women, 23.2% of heterosexual men, and 16.3% of gay or bisexual men had perpetrated physical or sexual IPV in the previous six months. In a study of HIV-positive men who have sex with men (MSM) in the midwestern USA [21], 78.4% had perpetrated psychological aggression, 39.2% had perpetrated physical assault, and 27.5% had perpetrated sexual coercion in their intimate relationships in the past year. 

### 2: Prevalence of HIV in people experiencing IPV

Ten studies from East Africa [31,63,78], India [20,23,58,89], Papua New Guinea [61], the USA [90], and Mexico [91] reported the prevalence of HIV in people experiencing IPV. All these studies found high prevalence rates, as shown in Table 4. 

**Table 4:** Prevalence of HIV by experience of IPV.

Region	Article	Population**^*a*^**	N	Age**^*b*^**	HIV prevalence**^*c*^**		
					IPV+	IPV-	p value
Asia	Chandrasekaran et al., 2007	WSM	245	15-45	67%	-	-
	Dayaprasad et al., 2010	WSM	636	16-45	P/S: 8%	P/S: 1%	<0.01
	Kulkarni et al., 2009	Trans	155	15-50	P/S: 37%	P/S: 3%	<0.01
	Lewis et al., 2008	WSM	415	15-60	P: 28.2%	P: 16.5%	P: 0.045
					S: 29.5%	S: 15.5%	S: 0.01
					V: 28.6%	V: 14.6%	V: 0.013
	Silverman et al., 2008	WSM	28139	15-49	0.73%	0.19%	-
North America	Sormanti et al., 2008	WSM	620	50-64	8.80%	3.30%	<0.05
	Ulibarri et al., 2010	Hispanic, FSW & WSM	300	33 (8.3)	9.50%	3.10%	0.02
Sub-Saharan Africa	Fonck et al., 2005	WSM	520	26 (6.8)	40%	-	-
	Makayoto et al., 2012	pregnant WSM	300	14-45	23.10%	14.90%	-
	Prabhu et al., 2011	WSM	2436	≥18	22.20%	19.30%	-

***^a^***Populations to which the estimates apply: WSM = women who have sex with men, Trans = transgendered, MSW = men who have sex with women, FSW = female sex workers. **^*b*^**Age range or mean age (standard deviation). **^*c*^**P = physical IPV, S = sexual IPV, V = verbal, psychological or emotional IPV or control.

### 3: Is experiencing IPV associated with HIV?

Many studies have been conducted internationally to determine whether IPV and HIV are associated. Findings of some of the quantitative studies are provided in Table 3 [42,55,56,60,76], which shows the results of statistical tests of IPV prevalence by HIV status, Table 4, which shows the results of statistical tests of HIV prevalence by experience of IPV [23,58,61,90,91], and Table 5 [14, 18, 20, 24-26, 31, 37, 39, 41, 44, 47, 49, 50, 55, 57, 63, 64, 66, 68, 72, 73, 76, 78, 82, 83, 87, 89, 91, 92, 94], which shows relative measures of association.

**Table 5:** Relative measures of association between experiencing IPV and HIV infection.

Region	Article	Population**^*a*^**	N	Age**^*b*^**	Bivariate Association**^*c*^**	Multivariable Association**^*d*^**
					(95% CI)	(95% CI)
					p value	p value
Asia	Chandrasekaran et al., 2007	WSM	245	15-45	P/S/V: OR=2.35 (1.39-3.96)	P/S/V: aOR^e^=0.57 (0.12-2.74)
	Ghosh et al., 2011	WSM	37781**^*f*^**	15-49		S^f^: aOR^e^=1.52 (0.75-3.07)
			22684**^*g*^**	15-49		S^g^: aOR^e^=2.63 (1.53-4.01)
	Silverman et al., 2008	WSM	28139	15-49		P: aOR^e^=0.89 (0.46-1.71)
						P+S: aOR^e^=3.92 (1.41-10.94) 0.01
						P-S: aOR^e^=1.53 (0.76-3.06)
Multiple**^*h*^**	Harling et al., 2010	WSM	60,795	15-49		P/S: aOR^e^=1.03 (0.94-1.13)
North		African American &				P+S: aOR=NS
America	Burke et al., 2005	WSM	611	≥18		0.78
	Greenwood et al., 2002	MSM	2881	≥18		P: aOR=1.5 (1.1-2.1)
						S: aOR=NS
						V: aOR=1.2 (0.87-1.6)
	McDonnell et al., 2003	African American & WSM	611	≥18		P: aOR=0.75 (0.52-1.07)
						S: aOR=0.84 (0.56-1.26)
						V: aOR=1.01 (0.71-1.42)
	Sareen et al., 2009	WSM	13928	≥20	P: OR=4.76 (1.53-14.80)	P: aOR=2.72 (0.95-7.83)
						P: aOR^e^=2.81 (0.88-8.97)
					P/S: OR=5.79 (2.10-15.97)	P/S: aOR=3.27 (1.30-8.24)
						P/S: aOR^e^=3.44 (1.28-9.22)
					P+S: OR=17.92 (3.62-88.74)	P+S: aOR=8.58 (1.65-44.52)
						P+S: aOR^e^=8.47(1.65-43.57)
	Ulibarri et al., 2010	Hispanic, FSW & WSM	300	33 (8.3)	P/S/V: OR=3.28 (1.16-9.30) 0.03	P/S/V: aOR^e^=NS
South America	Barros et al., 2011	non-Black/brown Brazilian & WSM	3193	15-49	V: PR=1.09 (0.7-1.8)	V: aPR=1.12 (0.7-1.8)
Sub-Saharan Africa	Dude et al., 2011	WSM	1582	15-49	P: OR=1.56 (0.62-3.95)	P: aOR^e^=NS
					S: OR=3.14 (1.16-8.53)	S: aOR^e^=NS
					<0.05	
					V: OR=4.95 (1.80-13.6)	V: aOR^e^=3.46 (1.34-8.78)
					<0.01	0.05
					P/S/V**^*i*^**: OR=1.98 (1.13-3.49) 0.05	P/S/V**^*i*^**: aOR^e^=1.86 (1.07-3.24)
	Dunkle et al., 2004	WSM	1366	16-44	P: OR^j^=1.56 (1.21-2.03)	
					S: OR^j^=0.88 (0.51-1.53)	
					P+S: OR**^*j*^**=1.66 (1.18-2.32)	
					0.001	
	Fonck et al., 2005	WSM	520	26 (6.8)	P/S: OR=1.8 (1.1-2.8)	P/S: aOR^e^=S
	Jewkes et al., 2006	WSM	1295	15-26	P/S: OR**^*j*^**=1.56 (1.08-2.23)	P/S: aOR^e^=1.16 (0.78-1.73) 0.45
	Jewkes et al., 2010	WSM	1099	15-26	P/S: OR**^*j*^**=1.80 (1.24-2.59)	
						P/S: aIRR^j^=1.51 (1.04-2.21) 0.032
		pregnant		-	P/V: OR=1.7 (1.3-2.2)	P/V: aOR=1.2 (0.9-1.6)
	Kiarie et al, 2006	WSM &	2836		<0.0001	0.1
		MSW				
					P/V: OR**^*k*^**= 4.8 (1.4-16)	
					0.01	
	Kouyoumdjian et al., 2013	WSM	10252	15-49	P/S/V: IRR=1.37 (1.08-1.75)	P/S/V: aIRR=1.55 (1.25-1.94) 0.000
					P: IRR=1.41 (1.09-1.82)	P: aIRR=1.62 (1.28-2.04)
					S: IRR=1.35 (1.03-1.76)	S: aIRR=1.58 (1.23-2.03)
					V: IRR=1.40 (1.09-1.80)	V: aIRR=1.70 (1.25-2.01)
	Makayoto et al.,	pregnant			P/S/V: OR=1.71 (0.9-3.3)	
	2012	WSM	300	14-45	0.08	P/S/V: aOR^e^=NS
	Maman et al., 2000	WSM	340	18-55		P: aOR=2.42 (1.20-4.87)
						S: aOR=2.39 (1.21-4.73)
	Mattson et al., 2009	WSM	8484	-		S: aOR=NS
	Ntanganira et al., 2008	pregnant WSM	600	18-47	P: OR=2.60 (1.84-3.68)	P: aOR^e^=2.38 (1.59-3.57)
	Ntanganira et al., 2009	pregnant WSM	387	29.4 (6.3)	P/V: OR=0.99 (0.66-1.50)	P/V: aOR^e^=1.06 (0.66-1.73)
						S: aOR^e^=0.82 (0.45-1.52)
	Pettifor et al., 2004	WSM	4066	15-24		0.55
						V: aOR^e^=1.00 (0.72-1.39)
						0.99
	Prabhu et al., 2011	WSM	2436	≥18	P/S: OR= NS	P/S: aOR=0.98 (0.75-1.28)
	Sa et al., 2008	WSM	1418	20-44	P/S: OR=1.01 (0.64-1.59)	P/S: aOR=0.72 (0.43-1.23)
						P/S**^*e*^**: aOR=0.72 (0.43-1.22)
	Shi et al., 2013	WSM	1904	15-49	P/S/V: OR=S	P/S/V**^*j*^**: aOR^e^=S
					<0.05	<0.05
	van der Straten et	WSM	921	21-40		P: aOR^e^=0.72(0.46-1.12)
	al., 1998					0.15
						S^l^: aOR^e^=1.89 (1.2-2.96)
						0.006
						S^m^: aOR^e^=1.50 ( 0.93-2.42) 0.099
	Were et al., 2011	WSM	3408**^*n*^**	≥18	P/S/V: OR=1.22 (0.98-1.53) 0.081	P/S/V: aOR^e^=1.33 (1.01-1.76) 0.043
					P/S/V: OR^o^=2.71 (1.26-5.82) 0.011	P/S/V: aOR^p^=1.62 (0.59-4.47) 0.35
		MSW	3408**^*n*^**	≥18	P/S/V: OR=1.34 (0.97-1.85)	P/S/V: aOR^e^=2.20 (1.40-3.44) 0.001
					P/S/V: OR^o^=2.09 (0.93-4.69) 0.07	P/S/V: aOR^p^=1.69 (0.62-4.64) 0.31

***^a^***Populations to which the estimates apply: FSW = female sex workers, WSM = women who have sex with men, MSM = men who have sex with men, MSW = men who have sex with women. **^*b*^**Age range or mean age (standard deviation). **^*c*^**Association between having experienced IPV and prevalent HIV infection, unless otherwise stated. P = physical IPV, S = sexual IPV, V = verbal, psychological or emotional IPV or control, S = significant, NS = not significant. **^*d*^**Association between having experienced IPV and prevalent HIV infection, adjusted for sociodemographic factors, unless otherwise stated. aOR = adjusted odds ratio, aIRR = adjusted incidence rate ratio, aPR = adjusted prevalence ratio, P = physical IPV, S = sexual IPV, V = verbal, psychological or emotional IPV or control, S = significant, NS = not significant. **^*e*^**Association adjusted for HIV risk factors, as well as sociodemographic factors. **^*f*^**Sexually-experienced model: sample includes all sexually-experienced women. **^*g*^**Couple-linked model: Sample includes all married women currently living with their husbands, where the women’s data could be linked to their husbands’ HIV test data. **^*h*^**Study conducted in Dominican Republic, Haiti, India, Kenya, Liberia, Malawi, Mali, Rwanda, Zambia and Zimbabwe. **^*i*^**IPV defined using total violence score, which was derived from a factor analysis of emotional, physical, and sexual violence data. **^*j*^**Association for IPV experienced more than once compared to once or not at all. **^*k*^**Association after participants notified their partners of their HIV test results. **^*l*^**Sexual IPV defined as a partner insisting on sexual intercourse. **^*m*^**Sexual IPV defined as a partner getting mad if sex was refused. **^*n*^**This N refers to 3408 serodiscordant couples, in 2299 of which the HIV-infected partner was female. **^*o*^**Association between IPV and incident HIV infection. **^*p*^**Association between IPV and incident HIV infection adjusted for HIV risk factors and sociodemographic factors.

Five qualitative studies reported data on the link between IPV and HIV. One HIV-positive woman in a 1992 study in Baltimore, Maryland, USA [38] stated that “she had become infected from her baby's father and that she had finally left him because she “got sick of him beating up on [her].”” Authors of a study about women with HIV in Alabama, USA [62] reported that “[t]he collective experience of most women was that domestic violence had played a crucial role in becoming HIV-positive…” In an interview in 2002, a Latina heterosexual woman in Los Angeles, USA, described how she had been infected by her husband who was “physically and emotionally abusive” [112]. In a 2008 to 2009 study in Canadian cities with Aboriginal HIV-positive women who had experienced sexual violence [81], a participant described how sexual IPV results in increased risk of HIV: “...The sexual violence affects um, they leave a huge scar and if it's several incidents of sexual violence...Like if the initial sexual violence that occurs escalates and also it affects your sexuality, it affects you sexually. It either closes you down or it manifests itself in perverse ways, sexually. You know? Um, and it affects your self worth and so all these things when you put them together make you vulnerable to HIV, all these things...” In contrast, in a study conducted with HIV-positive women on antiretroviral therapy (ART) in Kampala and Mbarara, Uganda [96], “...[HIV positive status was] mentioned as another stress, not a cause of violence itself.”

The results of quantitative studies that have explored the association between IPV and HIV vary significantly. The study results are grouped here according to whether they used cross-sectional data about prevalent HIV or longitudinal data about incident HIV. Those using cross-sectional data are grouped by region of study (with regions with the most data presented first) and whether an association was identified, then by the period when the study was conducted.

#### Cross-sectional data from multiple regions

In an analysis of DHS data from 2003 to 2007 on ever married women from 10 developing countries (Dominican Republic, Haiti, India, Kenya, Liberia, Malawi, Mali, Rwanda, Zambia and Zimbabwe) [44], there was no difference in HIV in multivariable models between those who had experienced physical or sexual violence and those who reported neither physical nor sexual IPV with their most recent partner, or between those who experienced physical and sexual IPV and those who experienced no sexual IPV with their most recent partner.

#### Cross-sectional data from sub-Saharan Africa

Three studies found a difference in HIV by IPV only in bivariate analysis. In men in a 2008 population-based study in Eastern Cape and KwaZulu-Natal, South Africa [49], there was an association between relationship control and HIV status: percentages of participants who were HIV-positive and HIV-negative, respectively, were 12.4% and 13.8% of those with low equity on the relationship control scale, 77.7% and 67.3% in those with mid equity, and 9.8% and 19.0% of those with high equity. Among 1295 sexually active women in Eastern Cape, South Africa from 2002 to 2003 [47], those who had had more than one episode of physical or sexual IPV were 1.56 times as likely to be HIV-positive in bivariate analysis. A 2006 study from Nairobi, Kenya of 2836 pregnant women and their partners [55] found that 37% of those who tested positive for HIV had experienced physical, psychological, or financial IPV compared with 26% of those who tested HIV-negative.

One study found a significant association between IPV and HIV only after adjusting for confounders. In HIV-discordant couples in East and Southern Africa from 2004 to 2007 [94], HIV-positive men and women were more likely to report physical, verbal or sexual IPV.

In four studies, IPV and HIV were significantly and positively related, including after adjusting for confounding variables. Women who attended an STI clinic in Nairobi, Kenya between 1996 and 1997 [31] who had experienced physical or sexual IPV were 1.8 times as likely to be infected with HIV as those who had not experienced IPV. In a study of 3422 women aged 15 to 24 in the Rakai District, Uganda from 2001 to 2003 [99], sexual coercion ever was associated with HIV infection in bivariate analysis, and this effect was modified by alcohol use before sex in the past year: women with a lifetime history of sexual coercion and no alcohol use had an OR for HIV of 1.51 and women with both sexual coercion ever and alcohol use had an OR of 2.28, compared with women with no sexual coercion ever and no alcohol use. HIV-positive participants in a 2006 study in Rwanda of 600 pregnant women [72] were 2.60 times more likely to report physical IPV in the last 12 months in bivariate analysis and 2.38 times as likely in multivariable analysis, compared with HIV-negative participants. In a study using Demographic and Health Survey (DHS) data on 1904 women in Kenya from 2008 and 2009 [87], IPV and HIV were consistently associated.

An association with HIV was identified only for certain types of IPV in three studies. HIV-positive women in a 1998 study in Kigali, Rwanda were more likely to report sexual coercion than HIV-negative women, specifically that their partner had ever insisted when she did not want to have sex (43% vs. 29%) and that their partner “gets mad” when sex was refused (46% vs. 29%). They were not more likely to report physical IPV (22% vs. 20%) [92,93]. In multivariable analysis, HIV was associated with insistence on sexual intercourse, but not with partner getting mad when sex is refused or with physical IPV. In a Soweto, South Africa study with pregnant women from 2001 to 2002 [26], HIV was associated in bivariate models with physical IPV, physical and sexual IPV, and with broad physical or sexual IPV (i.e. both physical and sexual abuse or one type of abuse at mid to high frequency), but not with sexual IPV alone, compared with limited or no physical or sexual IPV. Only the association between HIV and broad IPV remained significant in a multivariable model. HIV and IPV frequency were also related in bivariate analysis: the OR for HIV was 1.11 for low frequency IPV, 1.45 for mid frequency IPV, and 1.75 for high frequency IPV, each compared to no physical or sexual IPV (p=0.0007). In a 2005 population-based study of married women in Rwanda [25], bivariate analyses revealed significant and strong associations between HIV and emotional IPV and sexual IPV, respectively, as well as between HIV infection and a total violence score, but not between HIV and physical IPV. In multivariable models, HIV infection was significantly associated with emotional IPV and with the total violence score, but not with physical violence or sexual violence.

In a study in Tanzania, lifetime IPV but not recent IPV was associated with HIV. Women in a 1999 Dar es Salaam study [65] who were HIV-positive were more likely to report physical violence ever (52.2% vs. 28.3%) and sexual violence ever (44.1% vs. 23.0%) than HIV-negative women. Physical violence in the past three months was not, however, associated with HIV (16.4% vs. 9.3%) [64]. Women who were HIV-positive also had more violent events with their current partner than women who were HIV-negative (10.53 vs. 4.05). There was an interaction between age and IPV on HIV status, with an adjusted OR of 9.99 for those who experienced IPV for those under 30 compared with those 30 and older [65]. 

In a study in from 2005 to 2008 in Moshi, Tanzania, a significant association between IPV and HIV existed only in single women and not in women overall. There was no difference in HIV seropositivity by the experience of physical or sexual IPV in women accessing HIV VCT in Moshi, Tanzania from 2005 to 2008 [78]. In contrast, IPV and HIV were associated in single women in bivariate and multivariable analyses: 22.4% of single women who experienced IPV were HIV-positive compared with 15.1% who had not experienced IPV.

Six studies from Africa had null findings. In a population-based survey conducted from 2002 to 2003 in Moshi, Tanzania [82], the prevalence of physical or sexual IPV was 10.7% in women who were HIV-positive and 10.6% in those who were HIV-negative. In a 2003 national survey in South Africa with women aged 15 to 24 [76], women with HIV were not more likely to have low relationship control than HIV-negative women or to report forced sex with their most recent partner. An analysis of DHS data from 2003 to 2006 in Zimbabwe, Malawi, and Kenya with women who were ever married [66] found that spousal sexual violence was not associated with HIV in multivariable models. Lifetime verbal or physical IPV did not differ by HIV status in a 2006 study of 387 pregnant women in Kabutare, Rwanda [73]. A 2010 study in Kisumu, Kenya with 300 pregnant women [63] found that 23.1% of those who reported psychological, physical, or sexual IPV in pregnancy and 14.9% of those who didn’t report IPV in pregnancy were HIV-positive. Finally, in a study with 104 HIV-positive and 152 HIV-negative Black women in communities around Pretoria, South Africa [54], HIV status was not associated with relationship control, male-dominated decision making, or female-dominated decision making in bivariate analyses.

#### Cross-sectional data from North America

Five studies found a difference in IPV rates by HIV status in bivariate analysis only. HIV-positive women reported significantly higher levels of verbal abuse and physical abuse in the past year than did HIV-negative women in a study of African-American mothers of HIV-negative children in New Orleans, Louisiana, from 1994 to 1997 [51]. In a study of African-American women conducted from 1994 to 2001 in Los Angeles, California [95], HIV-positive women were more likely than HIV-negative women to report at least one incident of domestic violence involving physical contact at baseline and at two follow up points: the mean was 2.96 and 1.34 incidents at baseline, 1.70 and 0.44 incidents at six months, and 1.46 and 0.41 incidents at 12 months, respectively. In 113 Latina women in Los Angeles, California in 2004 [71], those who had experienced physical IPV in the last six months were significantly more likely to be HIV-positive. In 620 women aged 50 to 64 in New York City, New York, USA [90], 8.8% of those who had experienced IPV were HIV-positive, compared with 3.3% of those who had not experienced IPV. A 2004 to 2006 study in Tijuana and Ciudad Juarez, Mexico with 300 female sex workers with a current partner [91] found that HIV and emotional, physical or sexual IPV in the past six months were strongly related in bivariate analysis.

In three studies, HIV was associated only with certain types of IPV. In a 1996 to 1998 study in four US cities with 2881 MSM [41], IPV in the past five years did not differ by HIV status, with prevalence rates in HIV-positive and HIV-negative men of 43.1% and 40.1% for any IPV, 38.5% and 34.6% for psychological or symbolic IPV, 28.7% and 21.4% for physical IPV, 6.4% and 4.9% for sexual IPV, and 24.6% and 17.6% for multiple forms of IPV, respectively. In multivariable regression, HIV infection was associated only with physical IPV, with an adjusted OR of 1.5, but not with psychological, symbolic, or sexual IPV. In 13,928 women in the 2004 to 2005 US National Epidemiologic Survey on Alcohol and Related Conditions [83], physical or sexual IPV in the past year was strongly associated with HIV infection as compared with no IPV, and the risk of HIV attributable to past year IPV was 11.8%. Those who experienced physical IPV only in the past year, as compared with no IPV, were more likely to have HIV, but this association was no longer significant after adjusting for confounding variables. Having experienced both physical and sexual IPV in the past year, as compared with no IPV, was strongly associated with HIV infection in multivariable models. A study of 186 MSM in Vancouver, Canada revealed that HIV infection was not associated with experiencing physical abuse, but was positively associated with experiencing psychological abuse in bivariate analysis [15].

Data from the 1997 to 1999 WAVE Study of 611 women in Baltimore, Maryland, suggested that only more severe or frequent IPV was associated with HIV. There was no significant difference in the odds of IPV by HIV status in bivariate or multivariable analysis [18,68], including for emotional, physical, or sexual IPV. However, in those who experienced abuse, frequent abuse (i.e. three or more incidents) was associated with HIV status for physical abuse (82% in HIV-positive women compared with 73% in HIV-negative women) though not for sexual abuse (74% vs. 59%). This finding was supported by data from study interviews; HIV-positive women “tended to report more experiences of repeated abuse,” whereas HIV-negative women were more likely to discuss a “singular abusive event.” In an analysis of the 445 women with a current intimate partner [60], the frequency of recent and prior physical and sexual IPV was similar by HIV status: no IPV ever was reported by 28% of HIV-positive and 28% of HIV-negative women, past IPV only by 28 and 30%, current IPV only by 4% and 9%, and both past and current IPV by 38% and 33%, respectively.

In a study of pregnant women in New York, Connecticut, Florida, and North Carolina, USA from 1996 to 1998 [56], there was no difference in physical or emotional abuse with a main male partner by HIV status, with 5.1% of HIV-positive and 5.8% of HIV-negative reporting physical abuse and 14.5% of HIV-positive and 17.4% of HIV-negative reporting verbal or emotional abuse. 

#### Cross-sectional data from Asia

Four studies in India found a difference in IPV rates by HIV status in bivariate analyses only. In ever married women in Chennai and New Delhi, from 2000 to 2001 [42], those with HIV were more likely to have ever experienced forced or coerced sex and to have ever been hit, kicked, or punched by their husband than HIV-negative women. Significantly more women who experienced physical or sexual IPV were HIV-positive in a 2008 study of 636 women attending an HIV/STI clinic in Bangalore [58], compared with those who did not report any physical or sexual IPV. In a parallel study of 155 transgendered persons attending the same clinic in 2008 (sex and gender not specified) [23], those who had experienced sexual or physical IPV were similarly significantly more likely to be HIV-positive. In women accessing HIV voluntary counseling and testing (VCT) in Bangalore in 2005 [20], the OR for the association between physical, psychological, or sexual IPV ever and HIV was 2.35 in bivariate analysis. The association was no longer positive or significant in multivariable analysis. 

HIV infection was significantly related to physical, sexual, and emotional, but not financial IPV in a 2006 to 2007 study in Papua New Guinea with women accessing antenatal care or HIV VCT [61].

In two studies using data from the 2005 to 2006 Indian National Family Health Survey, a significant association between IPV and HIV was found only for certain subgroups of participants. One study [37] revealed that sexual violence was not associated with HIV infection in women who had ever been married, but was positively associated with sexual violence in currently married women. In a separate analysis [89], married women who reported both physical and sexual IPV perpetrated by their husbands had a higher HIV prevalence than those who reported no IPV. In contrast, there was no difference by HIV status in having experienced physical IPV without sexual IPV or having experienced any physical IPV, each compared with having experienced no IPV.

#### Cross-sectional data from South America

In women in Sao Paulo, Brazil in 2001 and 2002 [14], only more severe or frequent IPV was associated with HIV. Comparing women who were HIV-positive with women who were not suspected to be HIV positive, the HIV prevalence ratio was 1.09 for only psychological IPV, 1.48 for moderate IPV, 1.82 for severe and episodic IPV, and 3.12 for severe and recurrent IPV. In a multivariable model, only severe and recurrent IPV remained significantly associated with HIV, with a prevalence ratio of 1.91.

#### Longitudinal data about incident HIV

Four studies assessed the association between IPV and incident HIV infection in women in Africa. In 3422 women aged 15 to 24 in the Rakai District, Uganda, from 2001 to 2003 [99], HIV incidence was 1.6 per 100 person years in women with no sexual coercion and no alcohol use before sex and 2.3 per 100 person years in women with sexual coercion but no alcohol use before sex. This positive association was not significant in multivariable analysis. In 1099 participants in the 2002 to 2006 Stepping Stones cluster-randomized trial in the Eastern Cape [50], women with more than one episode of IPV were 1.80 times as likely to be infected with HIV compared to women with one or no episodes of IPV, and the adjusted fraction of HIV attributable to more than one episode of physical or sexual IPV was 11.9%. In 3408 HIV-discordant couples in a study in East and Southern Africa from 2004 to 2007 [94], physical, verbal, or sexual IPV was not significantly correlated with the risk of HIV seroconversion in female or male participants who were HIV-uninfected at baseline. In another analysis of data on women in the Rakai District, Uganda from 2000 to 2009 [57], incident HIV was associated with sexual, physical, or verbal IPV ever, sexual IPV ever, physical IPV ever, and verbal IPV ever, each compared with no IPV ever. In multivariable analysis, each of these associations remained significant, and the adjusted attributable fraction for IPV ever on HIV was 22.2%. Using data only for the past year, HIV was significantly associated with any IPV, physical IPV, and verbal IPV, respectively, but not with sexual IPV. IRRs for the association between IPV and incident HIV tended to be greater for models of longer periods of exposure, for severe vs. minor forms of IPV, and for those who experienced IPV more frequently, though none of these differences was significant. Further, sexual abuse in childhood or adolescence seemed to modify the effect of any IPV on incident HIV, with an IRR of HIV infection of 2.50 for those who experienced any IPV ever compared to no IPV ever in those with sexual abuse, and 1.22 in those with no sexual abuse.

### 4: How is experiencing IPV associated with HIV infection?

Various mechanisms have been explored to explain how IPV and HIV could be related. The association may be causal, i.e. IPV may cause HIV or HIV may cause IPV, non-causal, i.e. IPV and HIV may be correlated, or both causal and non-causal. Potential causal mechanisms include that sexual IPV may increase the risk of HIV infection through trauma to the vaginal or rectal mucosa; risky behaviours, whether voluntary or involuntary, may increase the risk of HIV; relative immune compromise in people experiencing IPV may increase the risk of infection; and HIV infection may lead to IPV. Experiencing IPV could also be associated with HIV in ways other than causing HIV infection, for example experiencing IPV could cause delayed diagnosis, less frequent disclosure of HIV status, or poor access to HIV care. A non-causal relationship would exist if partners who perpetrate IPV were more likely to be infected with HIV, as indicated by either relatively high HIV infection rates or high HIV risk behaviours, or if there were antecedent factors which increase risk to both IPV and HIV, such as adverse experiences in childhood or adulthood.

Only one study has assessed for a causal mechanism between IPV and HIV independent of any correlation due to male HIV status, by looking at the association between HIV and IPV in women while controlling for male partners’ HIV status. Using data on 20,425 husband-wife dyads in the 2005 to 2006 Indian National Family Health Survey-3 [24], there was a significant multiplicative interaction of exposure to husbands’ HIV infection and physical or sexual IPV on wives’ HIV infection after controlling for sociodemographic and HIV risk factors; the adjusted odds of wives’ infection based on husbands’ HIV infection was 7.22 times greater in the presence of IPV as compared with in the absence of IPV [24].

#### Sexual assault may increase the risk of HIV infection

In five qualitative studies, participants described how sexual IPV may lead to HIV infection. Women who were seeking services for abuse in 2002 in Johannesburg, South Africa [101] noted that IPV put them at direct risk of HIV infection through forced or coercive unprotected sex with a risky partner, and coercive sex was described by the authors as “an ongoing feature of these women’s sexual relationships rather than a one-time violation.” In a study of HIV-positive women in Alabama, USA in 2004 [62], participants described the issue of “being cut” or “dry” during forced sex as increasing the risk of HIV infection: “The vaginal tears and abrasions that occur during forcible sex are a major conduit for HIV infection…” In a study in Botswana with people working in women’s non-governmental agencies and police stations [108], a participant from Women Against Rape indicated that the increase in the number of raped and battered women each year “has serious implications for women, who are increasingly facing the risk of contracting HIV/AIDS because when these violent acts are performed, there is little or no evidence of condom use.” A woman in a study in Western Cape, South Africa [111] identified how sexual IPV can lead to HIV infection: "[gender-based violence] can play a role sometimes in HIV/AIDS, because men can rape women, and there are also fights that are taking place within the household, lastly men don't want to use condoms by force, so they end up infecting their partners." Another participant stated, “Also when one is raped she can get HIV” [111]. An HIV-positive Aboriginal woman in a study from 2008 and 2009 in Canadian cities [81] described a sexual assault which she felt led to her HIV infection: “…Um, my last sexual assault was um, with this black guy from Africa. That’s when I became HIV positive. My husband of 17 years left and I was grieving at the time, I was very vulnerable at the time that I met him. On my first date I, you know, same thing. Um, the first date was a sexual encounter except that um, I insisted that he use a condom if he was going to have sex with me because I didn’t feel that I, you know, wanted to be, wanted to feel safe that way. But during the sexual act he ripped the condom off and I was, I tested positive for HIV about 12 weeks later...”

#### Behaviours in those who experience IPV may increase HIV risk

People who experience IPV may be unable to negotiate safer sex practices with their partners, for example condom use, types of sexual acts, or frequency of intercourse. This could lead to an HIV-negative person having an increased risk of contracting HIV, or to an HIV-positive person having an increased risk of transmitting HIV. People who experience IPV may also voluntarily engage in sexual behaviours that increase risk for HIV, such as unprotected sex, having multiple partners, or using substances such as alcohol and drugs. These two mechanisms may be difficult to distinguish, given that many indicators such as unprotected sex may be the same. However, they differ in that women are making decisions regarding risky behaviours in the second mechanism; they are grouped together here.

Data from qualitative studies illustrate how an HIV-negative person experiencing IPV may be at increased risk of HIV infection because of an inability to negotiate safer sex. A woman with HIV who was interviewed between 1995 and 1996 in a midwestern city in the USA [110] experienced such extreme social isolation since she was “not allowed to socialize or leave the home” that she did not know about HIV/AIDS, thereby preventing her from taking measures to reduce her risk of HIV infection and from getting tested or being diagnosed in an early stage of her illness: “Hearing her diagnosis was the first time anyone had spoken to her about HIV or AIDS.” In a study in Chennai, India from 2000 to 2001 [102], participants stated that women have limited ability to “discuss [their partners’] infidelity, refuse sex, or negotiate condom use” because of the threat of violence, and further explained that “refusing to have sex with a nonmonogamous husband is not a viable HIV-preventive strategy for women.” A woman’s initiation of condom use was “seen as a sign of insubordination, or more commonly, as a sign of her infidelity; both are common triggers to violence.” In a 2003 study in Mbale District [53], Uganda, a female focus group participant stated “men never allow us to use condoms, if we suggest they beat us.” An HIV-positive woman in an informal settlement in Nairobi, Kenya in 2005 [12] described her inability to negotiate condom use because of the threat of violence by her partner: “I have on many occasions refused to have sex without condoms with my husband but he insists and threatens to beat me, therefore, I have to give in. I know the risks of re-infection and that is why I do not enjoy sex. But what can I do?” Women in a 2002 study in Johannesburg, South Africa [101] indicated that violence and threats of violence kept women from using risk reduction measures such as abstaining from sex, limiting their number of sexual partners, and using condoms, and that this may be due to their inability to communicate about subjects such as sex, condom use, infidelity, and HIV and other STIs. They reported that trying to negotiate safer sex prompted further IPV, leading to “a reciprocal relationship between risk for abuse and HIV.” Investigators in a study of HIV-positive women in Alabama, USA in 2004 [62] identified the participants’ lack of control over sexual risk: “[t]he women were often infected with STIs in abusive relationships. In retrospect, they realized that being infected with these “lesser” infections was a sign of being at risk of HIV/AIDS. Being diagnosed with a STI was a stark reminder of the men's sexual prerogative in having other sexual partners, of the men's control over condom use (virtually none was reported), and over the timing and frequency of sexual intercourse in relationships...” Participants also discussed their lack of control over condom use, with one participant reporting that her partner “…said it didn’t feel natural” and another saying her partner “…just refused.” In a study in Botswana [108], a participant from the legal aid and counselling centre Emang Basadi reported: “Most [women who seek help] are not in control of their sexual lives and, therefore, are at high risk of contracting and spreading HIV/AIDS.” 

Two studies describe how HIV-positive people experiencing IPV may be more likely to transmit HIV because they are unable to negotiate safer sex or they have risky sexual behaviours. In a 1992 study in Baltimore [38], one participant reported that when she asked her partner to use condoms, “[h]e got mad. Why? He asked me why? I said I'm just asking you. I didn't want to take it no further than that... I would have to have my mind straight to say what I have to say to him because he's the type of person to, he's not violent or nothing like that. It's just he gets mad about something, he gets real angry and then sometimes he will. We have fought and all that. I ain't going to try to get in with that.” A second participant continued to be sexually active with her HIV-negative partner for more than one year after her diagnosis, because of her fear of his violent reaction to her disclosure of HIV status [38]. At the time of the interview, this woman had known of her HIV status for just over one year and she reported continuing to be sexually active with her male partner, who had tested negative. In a study conducted from 2008 to 2009 in Canadian cities with Aboriginal HIV-positive women who had experienced sexual violence [81], a participant described finding it difficult to reject men and simultaneously fearing transmitting HIV: “...Like I was finding it very hard to reject men and, in a ways but um, I was rejecting them but feeling fear for rejecting them because some men don't like being rejected. They're very aggressive and they said when they want sex they, they go out and pursue it aggressively. But I found I was like um, always that fear of transmitting, always that fear...”

Another qualitative study shows how women who experience IPV may voluntarily engage in behaviours that could increase their risk of HIV infection. Women in a 2002 Johannesburg, South Africa study reported that the “neglect that abused women may feel in their relationships could encourage women to seek out other partners…” and that as a mechanism of coping with IPV, women may also “abuse substances or alcohol, which could result in risky sexual behavior…” [101].

There are also many quantitative studies that have examined the association between IPV and behaviours which could increase the risk of HIV infection, specifically condom use, sexual frequency, discussion of HIV prevention, number of sexual partners, commercial sex involvement, and substance use. 

There is substantial evidence from quantitative studies about the association between IPV and condom use, and six studies in the USA, Tanzania, and India found a statistically significant positive association. In a study of HIV-positive persons who were homeless or at severe risk of homelessness in Baltimore, Chicago, and Los Angeles, USA [45], unprotected sex in the past 90 days was associated with physical IPV in bivariate (OR=2.01, 95% CI 1.40-2.88) and multivariable (aOR=1.74, 95% CI 1.15-2.65) analyses. In HIV-positive persons in the US Risk and Prevention Survey [16], having experienced physical, sexual, or verbal IPV in the past six months was associated with unprotected sex in the past six months (OR=2.52, 95% CI 1.85-3.43, p<0.001; aOR=2.6, 95% CI 1.2-6.0, p<0.01). Physical or sexual IPV was associated in unadjusted models with having used a condom in the past 12 months (OR=1.47, 95% CI 1.03-2.09) in a population-based survey of women in Moshi, Tanzania from 2002 to 2003 [82]. In married women in the 2005 to 2006 Indian National Family Health Survey [89], IPV was associated with women’s lifetime condom use (p<0.001). Lifetime IPV was associated with unprotected sex in the past six months in HIV-positive crack-cocaine users in Atlanta and Miami from 2006 to 2010 [52], with an adjusted prevalence ratio of 1.46 (95% CI 1.12-1.90). In 304 HIV-positive women in Georgia and Alabama, USA [59], those who reported IPV were more likely to report inconsistent condom use in the past 30 days (aOR=2.91, 95% CI 1.15-7.42) and in the past six months (aOR=4.35, 95% CI 1.78-10.64), no condom use at the time of last intercourse (aOR=2.64, 95% CI 1.20-5.82), and having never used condoms in the past 30 days (aOR=3.49, 95% CI 1.30-9.39).

Five other studies found an inconsistent or null association between IPV and condom use, including studies in the USA and in countries in East and Southern Africa. In 425 women with a current male partner in Baltimore, USA [39], condom use at last sex was not significantly associated with having had one to 12 IPV events in the past year (aOR=1.43, 95% CI 0.78, 2.64), but was associated with 13 or more IPV events (aOR=0.60, 95% CI 0.38-0.95), compared to those reporting no events. In women in antenatal clinics in Soweto, South Africa from 2001 to 2002 [26], physical or sexual IPV was not associated with never using a condom (OR=1.09, 95% CI 0.86-1.37). Physical or sexual IPV was also not associated with having correctly used a condom at last sex with a main partner in sexually active women in 70 rural villages in Eastern Cape province in South Africa from 2002 to 2003 [47]: correct condom use was reported by 37.0% (95% CI 32.0-42.0) of 951 women who experienced IPV more than once and 38.7% (95% CI 35.1-42.4) of 344 women who experienced IPV once or not at all. In a longitudinal study of 3408 HIV-discordant couples in East and Southern Africa from 2004 to 2007 [94], unprotected sex with their study partner in the past month was associated with IPV in women (OR=1.98, 95% CI 1.59-2.45, p<0.001; aOR=1.86, 95% CI 1.46-2.37, p<0.001) but not in men (OR=1.26, 95% CI 0.81-1.98, p=0.305). In women aged 50 to 64 in New York City [90], IPV in the past two years was not associated with condom use (p>0.05), but was associated with forced sex without a condom in the past two years: 20.6% of those with IPV and 0.3% of those with no IPV reported forced sex without a condom. 

Studies in Mexico and the USA did not find an association between sexual frequency and IPV experience. In 300 female sex workers in Tijuana and Ciudad Juarez, Mexico from 2004 to 2006 [91], physical, sexual, or emotional IPV in the past six months was not associated with mean number of unprotected vaginal sex acts or mean number of unprotected anal sex acts with the partner, with unadjusted odd ratios of 1.01 (95% CI 1.00-1.03, p=0.07) and 1.08 (95% CI 1.00-1.18, p=0.09), respectively. Similarly, physical or sexual IPV in the past two years was not associated with vaginal sex in the past six months in women aged 50 to 64 in New York City, New York, USA [90] (p>0.05).

Four studies in South Africa, Rwanda, and the USA examined the association between discussing HIV prevention measures and IPV, with inconsistent results. Women in a study in Johannesburg in 2002 who were seeking services for abuse stated that their inability to discuss STI symptoms would prevent their partner from getting treated, which could increase their risk of acquiring HIV [101]. In 876 women in steady relationships in Kigali, Rwanda in 1988 [93], having ever negotiated condom use was associated with a male partner insisting to have sex when the woman does not want to in bivariate analysis (OR=2.90, 95% CI 2.08-4.05, p<0.001) and multivariable analysis (aOR=2.54, 95% CI 1.64-3.93, p=0.000), with partner gets mad when the woman refuses to have sex in bivariate and multivariable analysis (OR=2.77, 95% CI 1.84-4.18; aOR=2.44, 95% CI 1.55-3.84, p=0.000), and with physical violence in bivariate (OR=1.76, 95% CI 1.21-2.54, p<0.01) but not multivariable analysis (aOR=1.45, 95% CI 0.96-2.2, p=0.079). In a 2005 population-based study of married women in Rwanda, women in abusive marriages were less likely to have discussed HIV or HIV prevention with their husbands (OR=0.62, 95% CI 0.56-0.72, p=0.03) [25]. There was no association between IPV and negotiation of safer sexual practices in a study of 304 HIV-positive women in Georgia and Alabama, USA from 2006 to 2010 [59]: the adjusted OR was 1.80 (95% CI 0.68-4.79) for “did not ask partner to use condom” and 1.29 (95% CI 0.61-2.76) for “did not refuse sex without condom.”

There was a positive and significant association between having multiple partners and IPV in studies with women in South Africa and India, as well as in studies with men in the USA and East and Southern Africa. However, no significant association was found in studies of women in Tanzania or in East and Southern Africa. In women attending antenatal clinics in Soweto, South Africa in 2001 and 2002 [26], physical or sexual IPV was associated in bivariate analysis with having had five or more lifetime sexual partners (OR=1.77, 95% CI 1.42-2.22) and having a non-primary male partner (OR=1.72, 95% CI 1.37-2.16). In Houston, Texas from 2002 to 2003 [85], HIV-positive men who reported having ever experienced forced sex were also more likely to report a higher number of primary partners in the past 12 months, at a mean of 2.4 compared with a mean of 1.1 in those who did not report forced sex (p=0.001). In a longitudinal study of 3408 HIV-discordant couples in East and Southern Africa from 2004 to 2007 [94], having sex partners in addition to their study partner was not associated with experiencing IPV in women (OR=1.11, 95% CI 0.69-1.79, p=0.674) but was associated with experiencing IPV in men (OR=1.75, 1.11-2.76, p=0.016, aOR=2.57, 95% CI 1.61-4.10, p<0.001). In married women in the 2005 to 2006 Indian National Family Health Survey [89], IPV was associated with women’s lifetime sex partners (p<0.001). IPV was associated with having three or more partners in the past year in sexually active women in 70 rural villages in Eastern Cape province in South Africa from 2002 to 2003 [47]: 19.2% (95% CI 15.2-23.3) of those with more than one IPV episode and 7.1% (95% CI 5.5-8.7) of those with one or no IPV episodes reported having three or more partners. In a population-based survey of women in Moshi, Tanzania in 2002 and 2003 [82], those who had experienced physical or sexual IPV were not more likely to have had multiple sexual partners in the past three years (OR=1.61, 95% CI 0.99-2.63).

Two studies in South Africa between 2001 and 2003 and one study in the USA assessed the association between commercial sex involvement and IPV. In women attending antenatal clinics in Soweto, South Africa in 2001 and 2002 [26], having had transactional sex ever was associated with physical or sexual IPV (OR=1.83, 95% CI 1.39-2.41). In HIV-positive persons who were homeless or at severe risk of homelessness in Baltimore, Chicago, and Los Angeles, USA [45], exchanging sex for money, drugs or shelter was associated with physical IPV, with an OR of 2.53 (95% CI 1.83-3.48) in bivariate analyses and an adjusted OR of 1.85 (95% CI 1.25-2.73) in multivariable analyses. In sexually active women in 70 rural villages in Eastern Cape province in South Africa from 2002 to 2003 [47], IPV was associated with having transactional sex with a casual partner: 15.7% (95% CI 11.2-20.3) of those with more than one episode of IPV and 6.2% (95% CI 4.6-7.8) of those with one or no episodes of IPV, but not with transactional sex with a main partner: 28.3% (95% CI 22.7-33.9) of those with more than one episode of IPV and 19.9% (95% CI 17.0-22.8) of those with one or no episodes of IPV.

Drug and alcohol use were inconsistently associated with experiencing IPV in studies in Rwanda, the USA, South Africa, Uganda, Tanzania, and Mexico. In a 1988 study of 921 women with steady partners in Kigali, Rwanda [92,93], bivariate analysis revealed that a woman drinking alcohol was not associated with a male partner insisting to have sex when the woman does not want to (i.e. sexual coercion) (OR=1.08, 95% CI 0.81-1.45, p>0.05) but was associated with physical violence (OR=1.39, 95% CI 1.00-1.95, p<0.05). 

In HIV-positive persons participating in the HIV Cost and Services Utilization Study in the USA in 1998 [35], physical or sexual IPV was associated with binge drinking (OR=2.01, 95% CI 1.02-3.95; aOR=2.20, 95% CI 1.21-4.01), compared with being a nondrinker, and current drug dependence history (OR=2.93, 95% CI 1.66-5.18; aOR=1.85, 95% CI 1.08-3.20) compared with no history of drug dependence. In a 2001 to 2002 study of pregnant women in Soweto, South Africa [26], women who experienced both physical and sexual IPV or either type IPV at mid to high frequency were more likely to have an alcohol or drug problem then women who experienced one type of physical or sexual IPV at low frequency or no IPV (aOR=4.59, 95% CI 2.54-8.30). As noted, alcohol seemed to modify the effect of sexual IPV on HIV in 3422 women aged 15 to 24 in Rakai, Uganda from 2001 to 2003 [99], with women who used alcohol having a higher unadjusted OR and adjusted OR for prevalent HIV compared with women who did not use alcohol, though the differences were not statistically significant. In a population-based survey of women in Moshi, Tanzania in 2002 and 2003 [82], physical or sexual IPV was associated in unadjusted models with having used alcohol at least once a week in the last 12 months (OR=1.85, 95% CI 1.40-2.46). In 300 female sex workers in Tijuana and Ciudad Juarez, Mexico between 2004 and 2006 [91], physical, sexual, or emotional IPV in the past six months was not associated in bivariate analysis with having injected drugs in the past month (OR=1.10, 95% CI 0.60-2.03, p=0.76) or with having ever shared needles or injection equipment (OR=1.32, 95% CI 0.44-3.93, p=0.62). In HIV-positive persons who were homeless or at severe risk of homelessness in Baltimore, Chicago, and Los Angeles, USA [45], physical IPV was associated with ever having abused alcohol (OR=1.73, 95% CI 1.26-2.37; aOR=1.93, 95% CI 1.32-2.83), but not with drug use in the past 90 days or injection drug use ever in bivariate analysis, with ORs of 1.22 (95% CI 0.89-1.67) and 1.12 (95% CI 0.79-1.57), respectively.

#### People experiencing IPV may have relative immune dysfunction

Exposure to IPV could lead to relative immune suppression, e.g. due to the significant stress which may be associated with experiencing IPV, which could increase susceptibility to HIV upon exposure or lead to faster disease progression in people who are infected. This hypothesis has only been tested in three studies. Those with a high CD4 count (≥500) were more likely to have experienced physical or sexual IPV than those with a low CD4 count in bivariate and multivariable analyses (OR=2.59, 95% CI 1.12-5.96; aOR=2.36, 95% CI 1.01-5.49) in HIV-positive participants in the HIV Cost and Services Utilization Study in the US in 1998 [35]. Notably, there was no apparent trend in odds ratio by increasing CD4 count in bivariate or multivariable analysis, with adjusted odds ratios for CD4 count 50-199 of 0.93 (95% CI 0.46-1.88), for 200-499 of 0.85 (95% CI 0.56-1.29), and for ≥500 of 2.36 (95% CI 1.01-5.49), as noted, each compared with CD4 count 0-49. In HIV-positive women in a longitudinal study of 3408 HIV-discordant couples in East and Southern Africa from 2004 to 2007 [94], IPV was inversely associated with CD4 cell counts less than 350 (OR=0.64, 95% CI 0.49-0.84, p=0.001; aOR=0.72, 0.54-0.96, p=0.027), and the association was similar though not significant in those with CD4 cell counts between 350 and 499 (OR=0.79, 0.62-1.01, p=0.058; aOR=0.081, 95% CI 0.63-1.05, p=0.12), each compared to women of with CD4 cell counts 500 or greater. Experiencing IPV was not associated with WHO disease stage in women (OR=1.05, 95% CI 0.69-1.60, p=0.812) or men (OR=0.46, 95% CI 0.13-1.69, p=0.243), comparing those with Stage III and IV disease and those with Stage I and II disease. In HIV-positive men, experiencing IPV was not associated with low CD4 cell count in bivariate or multivariable models, compared to those with CD4 cell counts of 500 or more (<350: OR=0.59, 95% CI 0.33-1.06, p=0.076, aOR=0.66, 95% CI 0.37-1.18, p=0.164; 350-499: OR=0.69, 95% CI 0.39-1.22, p=0.202, aOR=0.66, 95% CI 0.36-1.19, p=0.166). In HIV-positive women in Texas, USA in 2010 [70], those who had experienced physical or sexual IPV ever had had more opportunistic infections than women who hadn’t experienced IPV, at a mean of 0.24 compared with 0.10 (p=0.0125). There was, however, no significant association between IPV and mean CD4 count, with a mean of 499.4 for women with a history of IPV and 549.4 for women with no history of IPV (p=0.08), between the Severity of Violence Against Women Scale scores and mean CD4 count (p=0.18), or between the Danger Assessment score and mean CD4 count (p=0.33).

#### HIV infection leads to IPV

The association between IPV and HIV could also be in the opposite direction, i.e. HIV infection could lead to IPV. The literature provides examples of several instances in which this may occur, including that disclosure of HIV status may lead to IPV, that people who are infected with HIV may deliberately try to infect their partners, that the illness or its treatment may cause violent behaviour, that partners of people with HIV may perpetrate IPV as an act of punishment or revenge, and that people with HIV may be less likely to leave violent relationships. 

IPV may occur as a consequence of disclosure of HIV status, in particular in HIV-discordant couples. In a 1992 study in Baltimore [38], a participant described her partner perpetrating IPV after she disclosed her HIV status to him: “One day, he kicked the TV... and knocked up all the furniture, and took soap and wrote “AIDS b___” on the mirror... Every time we would have an argument, that's what it would be, you know, “You b___, you gave me AIDS.” A 29 year old married HIV-positive female in a 1999 study in Dar es Salaam, Tanzania [64] recounted her experience of physical violence after disclosure: “When I informed [my partner] of the results there was endless violence in the house.” A female social worker in Western Cape, South Africa explained how HIV can lead to violence [111]: “The problem of HIV/AIDS... if you have been diagnosed, that means you brought it into the house, and then the next thing that will happen, I will beat you up because now you need to tell me with whom did you sleep that you brought this disease into the house.” In a 2004 study with HIV-positive women in Alabama, USA [62], participants reported that disclosing a diagnosis to an abusive partner “often led to an escalation in violence” and for some women, this occurred “through being blamed (usually erroneously) for infecting [their partners].” In a 2004 study of 26 women in Uganda who were in HIV-discordant partnerships and experiencing sexual IPV [28], a woman who was HIV-negative and whose partner was HIV-positive explained how she experienced sexual IPV after she and her partner were tested: “The moment we came back from the testing centre my husband was very annoyed and furious. After we received our HIV test results, he was positive and I was negative. He then started to drink heavily. He would come back home drunk and rape me.” Another participant, who was HIV-positive and whose husband was HIV-negative, described how her husband viewed HIV as a barrier to having children, and consequently forced her to have sex with him. She reported, “[h]is argument was that although he is HIV negative, he believes that he is HIV positive and that we should have at least two children before we die.” In a 2006 Lusaka, Zambia study [106], HIV-positive women identified that a woman coming home and telling her partner that she is infected is one of three main causes of domestic violence (which was commonly referred to as a husband beating his wife), along with the man coming home drunk and the woman refusing to have sex with her husband. One participant stated: “Men get annoyed if his wife tells him he [is] HIV-positive. Confusion in the house which may lead to divorce and domestic violence.” Authors of a study in Uganda with women on ART in Uganda reported how IPV followed one participant’s HIV testing [96]: “The [husband] was HIV negative and [she] tested positive, and then quarreling began in the home.” 

Several studies also described how a person might perpetrate sexual IPV in an effort to deliberately infect his or her partner. Women in Uganda in 2004 described how in relationships where the male partner was positive and the female partner was negative, sexual IPV was seen as an attempt by males to infect their female partners so they could blame women for their HIV infection, and to stop women from having extra-marital relationships [28]. In a 2004 study in Alabama, USA [62], an HIV-positive participant called Helen stated that her partner confessed to deliberately infecting her, saying “I only did it because I love you so much.” In a study of health workers and pregnant and nursing women in Zimbabwe [109], participants reported that during pregnancy, “[m]en totally refused, delayed, or tested for HIV but hid results from partners whom they forced to have sex” and provided “…accounts of HIV-positive men who did not disclose results to their partners and deliberately infected or attempted to infect their partners.” In 1995, an African-American woman in the midwestern USA described how she was intentionally infected with HIV [98]: “But what I really wanted to do is kill this guy that gave it to me. And that's why he left town, I believe. Because me and him used to fight a lot. And I believe that with me and him fighting a lot, he wanted to make sure that I got it. I just know it. I know that he knew that he was HIV and he wanted to give it to me. Because we used to have a lot of arguments and I felt that was the punishment he wanted to give me for all the years we were arguing.” In a 2007 study in New York City, USA with HIV-positive Latina women who had experienced IPV [104], a participant identified HIV infection itself as a form of abuse: “Being infected by men who know that they have the virus is not only a form of domestic violence but a crime.” In a study conducted from 2008 to 2009 in Canadian cities with Aboriginal HIV-positive women who had experienced sexual violence [81], a participant reported, “....Well I’ve been um, I was, I’ve been living with it since, since I met my partner, my son’s dad um, he knowingly infected me. Um, I didn’t find that out until like years later when he got sick and um...”

Another way that HIV infection could lead to IPV is that the illness itself or its treatment could lead to violent behaviour. In a study in New York, USA, with HIV-positive Latina women [104], participants “noted that their partner’s illness, further magnified by his depression and medications, could make him violent.” HIV service providers in a San Francisco study [100] described how HIV infection creates anxiety, low self-esteem, depression, and other mental health problems in many people which can make people more susceptible to self-directed violence and violence by or against others. In contrast, a woman in Uganda reported a decrease in her husband’s IPV perpetration as his illness progressed, though not when they both tested positive for HIV [96]: “She thought their illness would improve the relationship, but it did not. The main change came after the man began falling sick and lost energy, “after a while he was weak and did not have the energy to beat me.”” IPV did not significantly increase with initiation of home-based ART in a study of women with HIV in Uganda [13]; rates of physical IPV were 1% (3/459) in the three months before ART initiation and 2% (9/436) in the three months after program initiation, with an unadjusted OR of 3.20 (95% CI 0.94-10.9, p=0.063), though notably, 5 (1%) of the 436 women reported physical violence associated with program participation.

IPV as a consequence of disclosure of HIV status was also described as an act of revenge or punishment, and as a means of achieving the specific outcome of separation. HIV service providers in San Francisco stated that some clients perpetrate IPV in revenge against a partner who had infected them with HIV [100]. Women in a 2004 study in Uganda identified that in relationships where the female was positive and the male was negative, participants thought that male partners’ sexual violence might result from the males’ suspicion that females were infected because of infidelity, or as an attempt to force the partners to separate [28]. 

In several studies, participants expressed that their HIV infection prevented them from leaving violent relationships; in this way, HIV contributed to the continuation of IPV. HIV-positive Latina women in New York who had experienced IPV [104] expressed their feeling that very few men would want to be with a woman who was HIV-positive, and that having HIV meant they would have to endure IPV: “[T]he majority of women thought that their HIV status exiled them to abusive relationships.” HIV-positive MSM in Seattle in 2005 and 2006 described that HIV infection was an additional barrier to leaving violent relationships [75]. In a longitudinal study of 3408 HIV-discordant couples in East and Southern Africa from 2004 to 2007 [94], relationship dissolution occurred more often in women who were HIV-positive compared to women who were HIV-negative, however this was not the case for men: relationship dissolution occurred in 31.3% of HIV-positive and 22.5% of HIV-negative women and 20.7% of HIV-positive and 35.8% of HIV-negative men reporting IPV. An Aboriginal HIV-positive woman in a Canadian study in 2008 and 2009 with women with a history of sexual violence [81] reported that fear kept her in her relationship: “…Yeah like that's what kept me in the relationship, a lot of fear. And um, like he's threatened me many times...”

Several quantitative studies have also examined IPV that occurred subsequent to or as a consequence of HIV diagnosis, and studies in Kenya, Uganda, the USA, East and Southern African countries, and Nigeria found that IPV occurred, began, or worsened subsequent to HIV diagnosis. In a 1997 to 1999 study of postpartum women in Mombasa, Kenya [34], 31% (90/290) of HIV-positive women chose to inform their partner of their status, three of whom reported experiencing IPV as a result of disclosure. In 26 women in Uganda in 2004 who were in HIV-discordant partnerships and experiencing sexual IPV [28], sexual IPV either increased or started subsequent to HIV testing; two thirds of women reported sexual violence in their relationship prior to HIV testing, but they reported that violence increased at least twofold after their diagnosis, and for the other one third, sexual violence began subsequent to diagnosis. In women in Baltimore, Maryland, USA from 1997 to 1999 [39], being in an HIV-discordant partnership was associated with more frequent IPV, with an adjusted OR of 2.84 (95% CI 1.04-5.95) for those who experienced one to 12 IPV events and 2.51 (95% CI 1.26-5.00) for those who experienced 13 or more events in the past year, compared to those who experienced no events, for women who were in HIV-discordant partnerships compared to those in HIV-concordant partnerships. In a longitudinal study of 3408 HIV-discordant couples in East and Southern Africa from 2004 to 2007 [94], 36.9% of IPV reported at enrollment in HIV-infected women was assessed by the study staff as “definitely or probably related to the couples' learning of their serodiscordant HIV serostatus” compared to only 14.7% of IPV reports from HIV-uninfected women, with similar percentages in men (36.4% in HIV-positive and 13.0% in HIV-negative). In women seeking antenatal care in Nairobi, Kenya in 2006 [55], in the two weeks after receiving HIV test results, 0.9% (15/1638) reported IPV, and HIV-positivity was associated with IPV after HIV testing (aOR=4.8, 95% CI 1.4-16). In HIV-positive pregnant women in Lagos, Nigeria from 2006 to 2007 [30], abuse began after HIV diagnosis for 74.8% of the 428 women who reported IPV. In 25.9% (n=111) of women who reported IPV, the IPV occurred prior to HIV diagnosis, and of these women, 53.2% (n=59) reported worsening of IPV after diagnosis, 29.7% (n=33) reported no change, and 17.1% (n=19) reported no abuse since diagnosis. In bivariate analyses, factors associated with an increase in IPV after diagnosis were having an HIV-negative spouse (increased from 39.1% to 66.9%) and disclosure of HIV status (increased from 71.9% to 85.1%), however, in a multivariable model, the association with IPV persisted only for having an HIV-negative spouse (aOR=3.1, 95% CI 2.4-5.3). In HIV-positive persons attending a clinic in Pittsburgh, Pennsylvania, USA in 2010 [80], 7% of 56 participants reported that their partner became violent and/or physically attacked them upon disclosure of their HIV infection.

In contrast, in two studies in the USA, IPV did not start or worsen after HIV diagnosis or disclosure. In HIV-positive women in Baltimore in 1997 and 1998 [40], any and each of physical, sexual, and emotional IPV, respectively, occurred most commonly before the diagnosis with HIV: for 34% of 308 women any IPV occurred only before diagnosis, for 16% it occurred only after diagnosis, and for 17% it occurred both before and after diagnosis. Of note, those who were abused after learning of their status (N=86) reported more signs and situations involving drugs and alcohol (82.1%) compared with HIV-positive women who were abused only before learning about their infection (61.9% of N=105) and HIV-negative women (48.8% of N=199) [74]. In HIV-positive youth accessing services in New York City, USA, there was no significant association between HIV disclosure and physical IPV; the association and statistical test result were not provided [36].

#### IPV may affect HIV testing, HIV status disclosure, and access to HIV care

Experiencing or fearing IPV could affect the likelihood of HIV testing, of disclosing HIV status to a partner, and of accessing HIV care. 

With respect to an association between IPV and access to HIV testing, four studies had contrasting findings. A 32 year old married HIV-positive participant in a 1999 study in Dar es Salaam, Tanzania [64] identified fearing her partner’s reaction as a barrier to HIV testing and serostatus disclosure; when asked, “Why then did you decide not to tell him you are coming [to get tested]?” she responded, “I feared annoying him because he is very brutal.” In a 2003 study in Mbale District, Uganda [53], a female focus group participant explained that men usually react violently when women go for HIV testing, and view this as evidence of “prostitution” by the woman. Regarding her partner, another female participant stated: “he can ask me why I went for a [HIV] test and call me a prostitute and beat me.” In contrast, in women seeking antenatal care in Nairobi, Kenya in 2006 [55], IPV was not associated with reduced uptake of HIV counselling, HIV testing, or nevirapine use. A study in New York City, New York, USA of women aged 50 to 64 [90] found that a higher proportion of women who reported physical or sexual IPV in the past two years had been tested for HIV, at 64.7% compared with 42.5% in those who had not experienced IPV (p<0.05). 

Data from qualitative studies illustrate how fear of IPV or previous experiences of IPV may delay or prevent disclosure. In a 1992 Baltimore study with HIV-positive women [38], two participants described their failure to disclose their HIV status because of fear of IPV: “It took me maybe about three weeks before I told him. Cause, you know, I mean he had a right to know. But I was like scared. Even though he's not abusive physically, but that's what I thought. I thought he would get abusive because I didn't know. Had I known, I wouldn't keep it from him, but it's something that you think about before you do speak.” Another woman described her fear regarding disclosure, and conflict on the issue of disclosure with staff at a clinic where she accessed care: “I tried to tell him [her fiancé], in a way. I tell him when a commercial is on. I said, “What about if I told you that I am HIV positive?” He say “Yeah, I beat your ___....” I am scared to tell him now... They [the clinic] want me to come out and tell him. I keep trying to tell them, “I'll send him down here let y'all tell him. Don't say my name, cause that man is violent.”” In a study of HIV-positive women on ART in Kampala and Mbarara, Uganda [96], authors reported: “In Kampala, a woman who had suffered from ongoing physical and sexual violence from her husband feared to tell him. She had sores on her face and was trying to think of a way to make an excuse: “So I asked my friend, “So if I've got herpes zoster what am I going to tell the husband?” So we say that, we lie to him that you got a caterpillar on your face!”...The man believed the lie about the sores being from a caterpillar bite, which bought her some time before she eventually did tell him and he tested positive as well.”

Quantitative data also suggest that IPV or fearing IPV may lead to failure to disclose. In a 1988 Kigali, Rwanda study of women in steady relationships [93], not having discussed test results was associated in bivariate analysis with having a partner who gets mad when the woman refuses to have sex (OR=2.22, 95% CI 1.22-4.04, p<0.01), but not with a male partner insisting to have sex when the woman does not want to (OR=1.09, 95% CI 0.67-1.77) or with physical violence (OR=1.58, 95% CI 0.95-2.62). In pregnant women in Lagos, Nigeria from 2006 to 2007, 9.6% percent of women did not disclose their HIV-positive status to their partner for fear of stigma, rejection and possible abuse by their spouse [30]. Of 324 HIV-positive women in a Lusaka, Zambia study from 2001 to 2003 [84], disclosure of HIV status was not associated with verbal abuse (p=0.27) or physical violence from her partner (p=0.22).

Fear of violence may also impede HIV care. HIV-positive participants in a 2010 study in Gulu District, Uganda identified fear of violence as a barrier to accessing HIV care and ART for women in HIV-discordant and ART-discordant relationships [105]. They reported that their need for secrecy from male partners, due to fear of violence, resulted in avoiding HIV testing, hiding ART medications, and discontinuing ART. They also stated that the normalization of IPV in post conflict settings and the lack of legal protection for women experiencing IPV “buttresses these barriers to HIV care.” In HIV-positive women in Kampala and Mbarara, Uganda [96], two women who had experienced IPV reported that their husbands intervened with their HIV treatment: “One said her husband was throwing away her medication, saying that since they were both positive, they should die together (he tested, but had refused treatment). The other said the husband took her medication, presumably for himself because he would not go to the clinic.”

Considering quantitative data about access to HIV care, in a longitudinal study of 3408 HIV-discordant couples in East and Southern Africa from 2004 to 2007 [94], IPV was not associated with ART use in women (OR=0.24, 95% CI 0.09-0.61, p=0.003, aOR=0.42, 0.16-1.15, p=0.091) or in men (OR=0.46, 95% CI 0.13-1.69, p=0.243). In a multivariable analysis of data from a 2006 to 2010 study of inpatient HIV-positive crack-cocaine users in Atlanta and Miami, USA [52], lifetime IPV was associated with diminished use of HIV care in the past year, with a prevalence ratio of 0.87 (95% CI 0.75-0.99), but was also associated with current ART, with a prevalence ratio of 0.59 (95% CI 0.42-0.84). HIV-positive women accessing an urban HIV clinic in the USA in 2008 [46] who had experienced IPV in the past year were more likely to have missed gynecological appointments (aOR=3.5, 95% CI 1.65-7.21). In HIV-positive persons attending a clinic in Pittsburgh, Pennsylvania, USA in 2010 [80], those who had not experienced verbal, physical, or sexual IPV were significantly more likely to be taking HIV medication than those who had experienced IPV (93% vs. 66%, p=0.04), and 16% of participants reported being afraid to disclose their positive status to an intimate partner.

#### IPV perpetration is associated with HIV risk behaviours and HIV infection

Experiencing IPV would be correlated with HIV infection if those who perpetrate IPV have higher rates of HIV risk behaviours and are therefore more likely to be infected with HIV, which would, in turn, increase the risk of their partners. Relevant HIV risk behaviours include number of sexual partners, condom use, alcohol and drug use, and infection with other STIs. 

In a study in New York City, USA, IPV was associated with having a partner known or suspected to have HIV or who injected drugs; in women aged 50 to 64 [90], having experienced physical or sexual IPV in the past two years was associated with having had sex with a partner whom the participant knew or suspected was HIV positive or injecting drugs in the past six months (14.7% of those with IPV and 0.5% of those with no IPV, p<0.001).

Perpetrating IPV was inconsistently associated with less condom use in studies in South Africa and the USA. In a 2002 study with women experiencing IPV in Johannesburg, South Africa [101], many participants reported that their partners “…were opposed to condom use entirely, not just in the context of their intimate relationships but also within their casual relationships…” and were therefore having “concurrent, unprotected sexual relationships.” In HIV-positive persons in the US Risk and Prevention Survey from 1996 to 1998 [16], having perpetrated IPV was associated with unprotected sex in bivariate analysis (OR=1.69, 95% CI 1.15-2.5, p<0.01), as well as in multivariable analysis in women and in gay and bisexual but not heterosexual men, and in those who reported substance use but not those who did not. In a study in Baltimore, Maryland, USA from 2001 to 2005 of HIV-positive men who inject drugs [33], men who perpetrated IPV against their main female partner were more likely to have had unprotected sex with that partner (57% compared with 40% in those who did not perpetrate IPV, p<0.005) and to have had unprotected sex with HIV-negative or unknown status nonmain partners (16% compared with 6%, p=0.001). In contrast, the rates of IPV by unprotected sex with HIV-negative or unknown status main partners (18% compared with 12%, p<0.18) and with nonmain partners of any HIV status (28% compared with 20%, p=0.11), respectively, were not significantly different from those who did not perpetrate IPV. In a multivariable model, IPV perpetration against main female partners was positively associated with unprotected sex with main and nonmain HIV-negative female partners (aOR=1.68, 95% CI 1.02-2.77, p<0.05) and with unprotected sex with HIV-negative nonmain partners (aOR=2.69, 95% CI 1.10-6.56, p<0.05). 

Several studies have examined the association between perpetrating IPV and number of sexual partners, including two qualitative studies. In a study from 2008 to 2009 in Canadian studies with Aboriginal HIV-positive women who had experienced sexual violence [81], a woman described that her partner had had other partners: “...Cause he cheated on me. I found panties in his pants, he’d come home smelling like somebody else and all that lovely shit that happens when you love somebody so bad. You know and all the dirt you go through to - tryin’, you know, and you think that’s your worth, you know.” In a study in San Francisco, USA, HIV service providers indicated that male partners who cheat often perpetrate IPV [100]. They also described how Latino and African- American men who keep their bisexuality hidden from their female partners may be likely to perpetrate IPV when they “are confronted about their sexuality,” (i.e. about having other partners), and may also be at risk of HIV transmission. 

In quantitative studies in Rwanda, the USA, South Africa, Tanzania, Mexico, and Uganda, there was a positive association between IPV perpetration and having multiple partners. In a study in Baltimore, Maryland, USA from 2001 to 2005 with HIV-positive men who inject drugs [33], men who perpetrated IPV had a higher number of sex partners than men who did not report IPV perpetration, with means of 6.2 (standard deviation=10.1) and 3.3 (standard deviation=5.8), respectively (p=0.02). In a 2002 study with women experiencing IPV in Johannesburg, South Africa [101], 14 of 18 participants reported that their partners had concurrent sexual partners. Women in a population-based survey in Moshi, Tanzania from 2002 to 2003 [82] who had experienced physical or sexual IPV were more likely to have a partner with other wives or girlfriends in bivariate models (OR=1.99, 95% CI 1.48-2.67). Physical, sexual, or emotional IPV in the past six months was associated with having a spouse with another partner since the participant and the partner had been together (OR=2.55, 95% CI 1.46-4.45, p<0.01; aOR=2.45, 95% CI 1.34-4.82, p<0.01) in a 2004 to 2006 study with 300 female sex workers in Tijuana and Ciudad Juarez, Mexico [91]. In a 2005 population-based study of married women in Rwanda, women in polygamous marriages were more likely to have experienced emotional IPV (OR=4.75, 95% CI 2.76-8.17, p<0.01), physical IPV (OR=2.37, 95% CI 1.41-3.99, p<0.01), and sexual IPV (OR=1.74, 95% CI=1.02-2.98, p<0.01) ever [25]. In women accessing VCT in Moshi, Tanzania from 2005 to 2008 [78], women who had experienced physical or sexual IPV were more likely to report that their partners had other partners (OR=3.13, 95% CI 2.39-4.12). In HIV-positive women on ART in Kampala and Mbarara, Uganda [96], overall verbal violence score, physical IPV, sexual IPV, verbal IPV, and controlling behaviours were each associated with having a partner with other wives in bivariate analysis (p<0.05). 

A study in Rwanda found an association between IPV perpetration and being in a polygamous partnership only in bivariate analysis. In women in steady relationships in Kigali in 1988 [92,93], being in a nonmonogamous partnership was associated with a male partner insisting to have sex when the woman does not want to in bivariate (OR=1.57, 95% CI 1.16-2.12, p<0.01) but not multivariable analysis, with having a partner who gets mad when the woman refuses to have sex in bivariate (OR=2.05, 95% CI 1.40-3.02, p<0.001) but not multivariable analysis, and with physical violence in bivariate (OR=1.57, 95% CI 1.16-2.12, p<0.01) but not multivariable analysis (aOR=1.47, 95% CI 0.99-2.19, p=0.054).

Data from qualitative studies reveal that alcohol and drug use may be closely related to perpetrating IPV. Specifically, substance use may exacerbate IPV and may be used as a strategy for coping with IPV. In women experiencing IPV in Johannesburg, South Africa in 2002 [101], participants noted that alcohol makes IPV and demands for sex, including unprotected sex, worse. In a study with Hispanic, Spanish-speaking, heterosexual men in South Florida, USA [103], focus group participants described substance abuse, violence, and HIV as “branches of the same tree.” They identified substance abuse as a risk factor for relationship conflict which often results in domestic violence, and as a risk factor for risky sexual behaviours such as infidelity, unprotected sex, and prostitution. Domestic violence was also seen as a risk factor for substance abuse; victims would turn to substance abuse to deal with the trauma associated with victimization, and perpetrators would turn to substance abuse to deal with the trauma of having their partners leave them because of the abuse. One participant stated: “Drugs make you forget about protection [i.e. condom use]. Drugs, alcohol, that goes with the drugs also, put you in a state that you do not care about anything. Hmm, it creates domestic violence. Domestic violence creates separation, separation creates depression, depression creates alcoholism, as you said, and also creates prostitution, because you prostitute yourself. In reality they all are related, they go hand in hand with all of them. Being by one way or the other, but all having to do with it. And at the end the disease, AIDS, and then death.” Authors of a study about HIV-positive women and children in South Africa [27] described how alcohol use may have exacerbated IPV in a study participant: “The client indicates that her partner is verbally and physically abusive toward her. He is especially abusive to her when he is under the influence of alcohol.” 

IPV perpetration was inconsistently associated with alcohol and drug use in studies in Rwanda, the USA, South Africa, Uganda, and Mexico. In a 1988 study of women in steady relationships in Kigali, Rwanda [92,93], having a partner who drinks alcohol was associated with having a male partner who insists to have sex when the woman does not want to in bivariate (OR=1.99, 95% CI 1.33-3.01, p<0.001) but not multivariable analysis (aOR=1.60, 95% CI 0.98-2.63, p=0.061), with having a partner who gets mad when the woman refuses to have sex in bivariate (OR=2.71, 95% CI 1.50-4.91, p<0.001) and multivariable analysis (aOR=2.18, 95% CI 1.17-4.06, p=0.014), and with having a partner who perpetrates physical violence in bivariate (OR=3.75, 95% CI 2.07-6.78, p<0.001) and multivariable analysis (aOR=3.60, 95% CI 1.94-6.66, p=0.000). In a study in Baltimore, Maryland, USA from 1997 to 1999 [39], women who experienced IPV frequently, i.e. 13 or more events in the past year as compared to none, were more likely to have a partner with either an alcohol or drug use problem (OR=1.93, 95% CI 1.19-3.14) and to have a partner with both an alcohol or drug use problem (OR=3.10, 95% CI 1.41-6.80); these associations were not statistically significant for those who experienced one to 12 events compared to those who experienced no IPV events in the past year. In HIV-positive persons participating in the HIV Cost and Services Utilization Study in the USA in 1998 [35], binge drinking was associated with physical or sexual IPV perpetration in multivariable analysis (AOR=2.14, 95% CI 1.05-4.37) but not bivariate analysis (OR=1.99, 95% CI 0.98-4.03) and current drug dependence history was associated with IPV perpetration in bivariate and multivariable analyses (OR=3.90, 95% CI 2.35-6.48; AOR=2.50, 95% CI 1.48-4.23). In HIV-positive women on ART in Kampala and Mbarara, Uganda [96], partner’s drinking frequency was significantly associated with experiencing verbal violence (p<0.001) and controlling behaviour (p<0.001), and partner’s drunkenness frequency was associated with experiencing verbal violence (p<0.001) and overall violence score (p<0.001). In female sex workers in Tijuana and Ciudad Juarez, Mexico from 2004 to 2006 [91], physical, sexual, or emotional IPV in the past six months was associated with having a partner who had ever injected illegal drugs in bivariate analysis (OR=1.78, 95% CI 1.04-3.05), though this relationship was not significant in a multivariable model. 

In 300 female sex workers in Tijuana and Ciudad Juarez, Mexico between 2004 and 2006 [91], those with a partner who had had an STI in the past six months were not more likely to have experienced emotional, physical, or sexual IPV in the past six months (OR=2.42, 95% CI 0.85-6.92).

Eight studies examined the association between perpetrating IPV and HIV infection, including two studies in Tanzania, and India which found a positive association. In women accessing VCT in Moshi, Tanzania from 2005 to 2008 [78], women who had experienced physical or sexual IPV were more likely to suspect HIV in a current or past sexual partner (OR=1.68, 95% CI 1.28-2.21). As noted already, in husband-wife dyads in the Indian National Family Health Survey-3, the odds of HIV infection in 20,358 men whose wives were not HIV-infected was significantly higher in those who perpetrated IPV in their current relationship compared with those who didn’t perpetrate IPV (OR=1.94, 95% CI 1.02-3.69, p=0.043) [24], and this association persisted after adjusting for the husbands’ demographic and sexual risk factors (aOR=1.91, 95% CI 1.11-3.27, p=0.019) [24]. 

Six studies in Rwanda, Uganda, Nigeria, South Africa, and Canada found an inconsistent or null association between IPV perpetration and HIV status. In a 1988 study in Kigali, Rwanda [92,93], women with an HIV-positive partner were significantly more likely to report physical IPV in the couple in the past year: 33% of those with an HIV-positive partner and 18% of those with an HIV-negative partner reported having been beaten by their partners (p<0.01). In regression analysis [92], partner’s HIV status was associated with physical IPV in bivariate (OR=1.83, 95% CI 1.00-3.38, p<0.05) but not in multivariable analysis (aOR=1.78, 95% CI 0.86-3.65, p=0.12), with insistence on sexual intercourse when not wanted in bivariate (OR=1.74, 95% CI 1.00-3.03, p<0.05) but not multivariable analysis (aOR=0.97, 95% CI 0.45-2.1, p=0.95), and with having a partner who gets mad when the participant refuses sex in bivariate (OR=1.01, 95% CI 0.44-2.33, p=0.97) but not multivariable analysis (aOR=1.50, 95% CI 0.93-2.42, p=0.099). In HIV-positive women on ART in Kampala and Mbarara, Uganda [96], partner’s HIV status was not associated with any form of IPV. In HIV-positive pregnant women in Lagos, Nigeria in 2006 and 2007 [30], experiencing physical, sexual, or emotional/psychological IPV was associated with having a partner who was HIV-negative (OR=1.87, 95% CI 1.26-2.77) or whose status was unknown (OR=1.80, 95% CI 1.05-3.10), compared to having a partner who was HIV-positive. In 866 males in Kampala, Uganda in 2007 and 2008 [32], those who were HIV-positive were more likely to have perpetrated psychological and physical IPV in the past 12 months (OR=2.50, 95% CI 1.27-4.93; aOR=2.35, 95% CI 1.00-5.54), but not psychological IPV (OR= 1.34, 95% CI 0.71-2.51, aOR=1.41, 95% CI 0.94-2.11) or psychological or physical or sexual IPV (OR=1.74, 95% CI 0.89-3.40, aOR=1.85, 95% CI 1.09-3.15). In a 2008 population-based study in Eastern Cape and KwaZulu-Natal provinces in South Africa with 1229 men [49], HIV status was associated with ever having perpetrated more than one episode of physical IPV in bivariate (39.3% of HIV-positive and 28.7% of HIV-negative, p=0.003) and multivariable models (aOR=1.50, 95% CI 1.04-2.17, p=0.03), but not with ever having perpetrated sexual IPV in a multivariable model (aOR=0.96, 95% CI 0.60-1.53, p=0.848). There was an interaction between age and perpetration of physical IPV on HIV: in men younger than 25, having perpetrated more than one episode of physical IPV, compared to one or no episodes, was associated with HIV (aOR=2.08, 95% CI 1.07-4.06, p=0.03), whereas in men 25 and older, having perpetrated more than one episode of physical IPV was not associated with IPV (aOR=1.21, 95% CI 0.78-1.87, p=0.39). A population-based study of 186 MSM in Vancouver, Canada [15] revealed no difference in the perpetration of physical abuse by HIV status (r=0.02), whereas psychological abuse was perpetrated more frequently by HIV-positive men (r=0.16, p<0.05). 

#### Antecedent experiences may increase risk for both IPV and HIV infection

Three studies described how adverse experiences in childhood or adulthood could affect IPV and HIV. In a study from 2002 to 2003 in Ohio, USA, of 24 women with HIV who were 45 or older [107], 11 women “reported that mental health issues associated with childhood sexual abuse, domestic violence, and/or life crises contributed to their vulnerability to HIV,” and eight of these 11 women reported domestic violence from their husbands or boyfriends. In HIV-positive MSM in Seattle, Washington, USA in 2005 and 2006 [75], interviews revealed that “the same factors predisposing [participants] to acquire HIV also may have placed them at risk for victimization by a relationship partner…,” including childhood abuse and neglect, involvement in street life, commercial sex work, legal problems, the belief that they would not live long lives, poverty, and rejection based on disclosure of sexual orientation. The authors stated that participants also might be more likely to become involved in violent relationships because of their HIV infection, due to factors including infection-related stigma, social isolation and an intense fear of being alone, as well as “inadequate screening of potential partners due to an attentional bias towards HIV-status and away from other important characteristics, fear about the lack of availability of suitable dating partners, interpreting aggressive or jealous behaviors as benign or positive, and a strong desire for an idealized romantic relationship from a partner who can serve as “protector” against the adversity in a man’s life.” In a study conducted in several Canadian cities from 2008 to 2009 with Aboriginal HIV-positive women who had experienced sexual violence [81], authors explained that “…[m]any of the same factors implicated in Aboriginal women's exposure to violence were also responsible for their heightened exposure to HIV,” including “addictions, involvement in the sex trade and difficulties setting sexual boundaries or negotiating safer sex with men.”

### 5: What is the evidence regarding interventions to prevent IPV and HIV?

In a 2002 study with women experiencing IPV in Johannesburg [101], participants highlighted the need to involve men in interventions, given women’s limited ability to implement risk reduction strategies in their intimate relationships, and some women advocated financial independence as a possible solution to their abusive relationships.

Only four interventions were identified by this search. Two studies showed promising results with respect to the prevention of IPV [48,79], though not of incident HIV infection, and one showed promising results in terms of attitudes toward and intention to perform HIV-IPV risk reduction behaviours [113].

A randomized controlled trial was conducted with 1416 women and 1360 men aged 15 to 26 years in 70 rural villages in the Eastern Cape province in South Africa, where villages were randomized to Stepping Stones, a 50 hour participatory program to build knowledge, risk awareness, and communication skills and to stimulate critical reflection, or a three hour program on HIV and safer sex [48]. The intervention resulted in no significant difference in HIV incidence in the two groups at two years, in IPV perpetration at 12 months, or in rape or attempted rape at 12 or at 24 months. There was a non-significant difference in men reporting IPV perpetration at two years: 6.2% in the intervention and 9.6% in the control group. There was a significant decrease in transactional sex with a casual partner and problem drinking at 12 months, but not for either of these outcomes at 24 months, and not for ever having misused drugs at 12 or 24 months. There was no difference in women in the intervention and control groups with respect to behaviour change.

In a study which randomly assigned communities to the immediate intervention of a microfinance program combined with a gender and HIV training curriculum or to this intervention three years later, which was conducted between 2001 and 2005 in Limpopo Province in South Africa with low income women between the ages of 14 and 35 [79], physical and sexual IPV in the past year was reduced by 55% in women in the intervention compared with those in the control group, and this reduction remained significant in a multivariable model. There was no significant reduction in controlling behaviours by participants’ partners, on unprotected sex with a non-spousal partner in household co-residents, on the rate of unprotected sex at last intercourse with a non-spousal partner, or HIV incidence in community members.

A randomized controlled trial was performed with 515 female users of outpatient substance abuse treatment programs in the USA between 2004 and 2005 [19] to assess the effect of a five session HIV and STI safer sex skills building group, compared with a one session didactic HIV prevention education program. There was no significant association between the intervention and the relationship control or the decision-making dominance subscale, respectively, of the Sexual Relationship Power Scale at three months after the intervention, and in a mediation analysis, neither subscale mediated the positive effect of the intervention on reducing unprotected sex episodes at 6 months.

A pilot study of an HIV-IPV risk reduction intervention for Spanish-speaking Latinas was conducted in Detroit, Michigan, USA with 31 women [22]. The intervention consisted of two modules with the underlying themes “Are you in a healthy and safe relationship?” and “Keeping yourself healthy and safe,” and small group discussions related to HIV-IPV informational decision making and skills building activities. Between baseline and one month post-intervention, there were significant changes in attitudes toward HIV-IPV risk reduction behavior and intention to perform HIV-IPV risk reduction behavior, but not in subjective norms, i.e. perceived social pressure to adopt HIV-IPV risk reduction behaviour or in perceived control, i.e. perceived ability to perform HIV-IPV risk reduction behavior.

## Risk of Bias in Studies

Regarding quantitative studies, only a small proportion were population-based [15,24,25,27,32,37,41,44,47-50,54,57,66,76,82,83,87,89,99] while for remaining studies, participants were recruited from health care settings, community-based organizations or service providers, or existing research studies which were not population-based [12-14, 16-23, 26-31, 33-36, 39, 40, 42, 43, 45, 46, 51, 52, 55, 56, 58-65, 67-75, 77, 78, 80, 84-86, 88, 90-98].

Very few quantitative studies included any longitudinal component [13,19,22,48,50,57,79,94,95,99], and even fewer assessed incident HIV infection [48,50,57,79,94,99]. In studies of the association between IPV and HIV in which multivariable analysis was conducted, most adjusted for HIV risk factors [20,25,26,31,37,44,47,63,72,73,76,83,87,89,91-94,99].

## Discussion

This review identifies a high prevalence of experiencing IPV in people with HIV across regions, and very little data regarding the prevalence of perpetrating IPV in people with HIV. In most studies, the association between experiencing IPV and HIV infection was positive and statistically significant in unadjusted analysis. Some studies found a trend between intensity of IPV experienced (in terms of severity or frequency or both) and prevalence or risk of HIV infection [14,18,26,57,68], though this association was not always statistically significant. 

Many studies also identified a significant positive relationship between IPV and HIV in adjusted analysis, however, these findings were inconsistent, with no apparent pattern regarding populations of focus, geography or the period of study. The methodological issues in many studies, including the lack of control for correlations, the lack of longitudinal data to appropriately model this association, and the potentially inappropriate adjustment for mediating variables, further contribute to a lack of clarity regarding whether this association is causal.

In assessing data on how IPV and HIV are related, we found evidence that the association is bidirectional and that there may be both causal and non-causal mechanisms occurring; this contributes to a more sophisticated and detailed classification of potential mechanisms than what has been described in previous reviews [3,4]. Specifically, qualitative data from diverse populations illustrated how sexual assault could lead to HIV infection, experiencing IPV could increase HIV risk behaviours, HIV infection could lead to IPV, IPV perpetration and HIV risk or infection could be correlated, and adverse experiences in childhood and adulthood could lead to both IPV and HIV. There were few studies which assessed the quantitative association between experiencing IPV and HIV risk behaviours and between perpetrating IPV and HIV risk behaviours or HIV status; those which we identified were inconsistent in their findings. There were no quantitative data to support the hypothesized mechanism that sexual IPV increases HIV infection through genital injury and minimal evidence regarding whether people experiencing IPV may have relative immune compromise. With respect to other associations, experiencing IPV and HIV testing, disclosure, and care were also inconsistently associated. 

There are several notable limitations of individual studies, across studies, and of this analysis. Regarding individual studies, with few exceptions, quantitative studies were cross-sectional, which precludes determination of the temporal association and therefore of the causal nature of the association between IPV and HIV and between IPV and potential mediators of this association. In most studies of the association between IPV and HIV, multivariable analysis adjusted for HIV risk factors, which is appropriate if HIV risk factors function only as confounders but not if they mediate the association between IPV and HIV (i.e. are on the causal pathway between IPV and HIV); adjusting for mediators would lead to an underestimate of any association and potentially to Type II error, i.e. failing to detect an association which truly exists. All but one study [24] examined only a single hypothesized mechanism to explain the association between IPV and HIV, without controlling for other mechanisms, which prevents an understanding of whether the association between IPV and HIV is causal or non-causal or both, and of the relative importance of various hypothesized pathways. Small sample sizes of many studies, in particular in subgroup analyses, may be associated with inadequate power and Type II error.

Across studies, there was variability in how IPV was defined, specifically, the types of IPV measured and the instruments used to measure each type of IPV, the level of IPV considered to constitute exposure, e.g. experiencing IPV more than once [26,47,49,50] or at all, as well as in the period of exposure studied, e.g. exposure to IPV in the past three months or ever; in the populations studied, e.g. MSM or pregnant women; and in the settings where research was conducted, e.g. population- or clinic- based. These differences limit the comparability of results, and importantly, it is unclear whether the heterogeneity in findings may be due, at least in part, to issues of definition and measurement or to a modification of the association between IPV and HIV by geographic or cultural context, or sociodemographic or behavioural factors [37,57,65,78,99].

Regarding this review, in our search, we included only studies which assessed or described the association between IPV and HIV infection, and for feasibility reasons we excluded those which looked exclusively at IPV and HIV risk factors. Many of the studies we examined assessed both HIV status and HIV risk factors, and there is no reason to suspect a systematic difference between studies which assessed only HIV risk factors and studies which assessed HIV risk factors and HIV status. Given this, our review should present valid, though not comprehensive, data regarding the associations between IPV and HIV risk factors. Also, given *a priori* concerns regarding the heterogeneity of the data in quantitative studies, we did not perform any meta-analysis.

Strengths of this analysis include the comprehensiveness of the search, which included gray literature and qualitative data, as well as studies from around the world and which focus on various subpopulations; this allows for a summary of all relevant data on the association between IPV and HIV without presupposing that geography or population modifies the association. In terms of methods, two reviewers independently reviewed all records and articles, and we completed a full review of any records considered eligible by either of the reviewers at the first stage of the review (i.e. abstract review), in order to optimize the inclusion of relevant articles.

This review identifies information that is relevant to public health policy and practice, notwithstanding the aforementioned limitations within and across studies. Experiencing IPV and HIV infection were associated in most unadjusted analyses, which indicates that in general, people experiencing IPV are more likely to have HIV. This information supports the need for initiatives such as screening for IPV as part of HIV VCT and primary care services. Screening could lead to referral for those who are experiencing or who have experienced IPV to counselling, legal assistance, and other community services. Screening could also contribute to the diagnosis of more people with HIV, including persons who might not otherwise be considered to be at increased risk. If the IPV-HIV association is causal, then incorporating information about this association into programs could also lead to the primary prevention of HIV.

Regarding future research, studies should address outstanding four fundamental issues. First, it remains unclear which type of IPV is relevant to HIV infection, so studies should measure and analyze data on all forms of IPV [114] and assess their respective associations with HIV; in this way, for studies in this field, IPV could be defined in a way which is most relevant to the association with HIV, and exposure misclassification bias could be mitigated. Second, studies should assess the period of exposure which is relevant for the association between IPV and HIV, e.g. lifetime exposure compared with recent exposure, and determine how long the risk of HIV may remain increased subsequent to exposure, which of course would require longitudinal data from large cohort studies over many years. Third, studies should assess which instruments are optimal for measuring IPV, recognizing that this may vary depending on social and geographical factors. Fourth, researchers should collect data on potential mechanisms (as outlined above) using longitudinal studies so that it is possible to determine which mechanisms lead from IPV to HIV infection. For each of these fundamental issues, qualitative research could also provide valuable information, as demonstrated in this review, and much of this research could be conducted within the context of intervention studies. 

There is also a clear need for evidence regarding interventions to prevent IPV and HIV, which could be assessed while simultaneously addressing the fundamental questions identified above. Interventions should build on the promising trials identified in this review, which suggest ways to prevent IPV [48,79] and change attitudes about IPV-HIV risk reduction behaviours [22]. They should have a long enough follow up period to capture long-term impacts of experiencing IPV (if it emerges that IPV exposure increases risk of HIV in the long-term or if there is a lag between exposure and an increase in risk) as well as enough incident cases of HIV for the study to be adequately powered.

## Supporting Information

Checklist S1 **PRISMA checklist of items to include when reporting a systematic review.**
(DOC)
